# A retinoic acid receptor β2 agonist attenuates transcriptome and metabolome changes underlying nonalcohol-associated fatty liver disease

**DOI:** 10.1016/j.jbc.2021.101331

**Published:** 2021-10-21

**Authors:** Xiao-Han Tang, Marta Melis, Changyuan Lu, Andrew Rappa, Tuo Zhang, Jose Jessurun, Steven S. Gross, Lorraine J. Gudas

**Affiliations:** 1 Department of Pharmacology, Weill Cornell Medical College of Cornell University, New York, New York, USA; 2 Genomics Resources Core Facility, Weill Cornell Medical College of Cornell University, New York, New York, USA; 3 Division of Anatomic Pathology, Department of Pathology and Laboratory Medicine, New York Presbyterian Hospital, Weill Cornell Medical College of Cornell University, New York, New York, USA

**Keywords:** nonalcoholic fatty liver disease, retinoic acid receptor, liver steatosis, fructose metabolism, vitamin A, DEG, differentially expressed gene, DIO, dietary-induced obesity, GSH/GSSG, glutathione/glutathione disulfide, HFD, high-fat diet, HSC, hepatic stellate cell, NAFLD, nonalcohol-associated fatty liver disease, NASH, nonalcohol steatohepatitis, RA, retinoic acid, RARβ2, retinoic acid receptor β2

## Abstract

Nonalcohol-associated fatty liver disease (NAFLD) is characterized by excessive hepatic accumulation of fat that can progress to steatohepatitis, and currently, therapeutic options are limited. Using a high-fat diet (HFD) mouse model of NAFLD, we determined the effects of the synthetic retinoid, AC261066, a selective retinoic acid receptor β2 (RARβ2) agonist, on the global liver transcriptomes and metabolomes of mice with dietary-induced obesity (DIO) using genome-wide RNA-seq and untargeted metabolomics. We found that AC261066 limits mRNA increases in several presumptive NAFLD driver genes, including *Pklr*, *Fasn*, *Thrsp*, and *Chchd6*. Importantly, AC261066 limits the increases in the transcript and protein levels of KHK, a key enzyme for fructose metabolism, and causes multiple changes in liver metabolites involved in fructose metabolism. In addition, in cultured murine hepatocytes, where exposure to fructose and palmitate results in a profound increase in lipid accumulation, AC261066 limits this lipid accumulation. Importantly, we demonstrate that in a human hepatocyte cell line, RARβ is required for the inhibitory effects of AC261066 on palmitate-induced lipid accumulation. Finally, our data indicate that AC261066 inhibits molecular events underpinning fibrosis and exhibits anti-inflammatory effects. In conclusion, changes in the transcriptome and metabolome indicate that AC261066 affects molecular changes underlying multiple aspects of NAFLD, including steatosis and fibrosis. Therefore, we suggest that AC261066 may have potential as an effective therapy for NAFLD.

Nonalcohol-associated fatty liver disease (NAFLD), which is defined as the accumulation of intrahepatic triglycerides without excessive alcohol intake and is usually associated with obesity, has become a primary cause of chronic liver disease. NAFLD can progress through histologically and clinically defined stages to nonalcohol steatohepatitis (NASH) or liver cirrhosis (1). In the United States, the number of NAFLD cases is projected to reach over 100 million in 2030, and 27% of adult NAFLD cases progress to NASH (2). Excessive accumulation of lipids in the liver induces liver stress and injury, resulting in the fibrogenesis and inflammation often observed in NASH (3). Despite an emerging NAFLD health crisis worldwide, to date there is no single FDA-approved therapy for preventing and/or treating NAFLD other than dietary intervention, weight loss, and medications for insulin resistance and hyperlipidemia (4). Therefore, it is crucial to identify and target the underlying molecular mechanisms that cause NAFLD to find a novel therapy. Here we explore a vitamin A agonist that defines a potential therapeutic approach.

Carotenoids and rerinoids, including vitamin A (retinol) and its metabolites, such as all-trans retinoic acid (RA), exert regulatory functions on multiple physiological processes (5, 6), including lipid metabolism and hyperglycemia control (6, 7, 8). Retinoic acid receptors (RARs α, β, and γ subtypes) are transcription factors that heterodimerize with retinoid X receptors (RXRs, α, β, and γ subtypes) and bind the endogenous agonist, RA, to regulate gene expression (9). In humans, histological stages of NAFLD, including mild and severe steatosis, NASH, and hepatocyte necrosis, show a strong inverse correlation with hepatic retinol levels (10). Hepatic retinoic acid levels are significantly lower in human NAFLD samples than in normal liver samples (11), and we also showed an inverse correlation between steatosis and hepatic retinol and retinyl palmitate levels (12). RA, a vitamin A metabolite and the endogenous agonist for all three RARs α, β, and γ, attenuated diet-induced liver steatosis in mice, indicating that activation of retinoic acid signaling could be novel therapy for NAFLD (6). We showed that the synthetic retinoid AC261066 (13) corrected hyperglycemia in type 2 diabetes mouse models, limited hepatic lipid accumulation, and prevented early hepatic fibrogenic events in a high-fat diet (HFD)-induced NAFLD mouse model (14, 15). However, we did not explore the molecular mechanisms involved in AC261066’s actions in depth in our prior work. Here, in addition to delineating the effects of AC261066 on the physiology, transcriptome, and metabolome in a related HFD-driven NAFLD mouse model, we establish a causal role for RARβ in regulating lipid metabolism and demonstrate that AC261066 acts through RARβ. These novel and important data suggest that AC261066 could be a useful drug for the treatment of NAFLD.

## Results

### HFD (high-fat diet) treatment induces increases in multiple transcripts involved in NAFLD pathogenesis and AC261066 limits/prevents these increases

**Figure 1:**
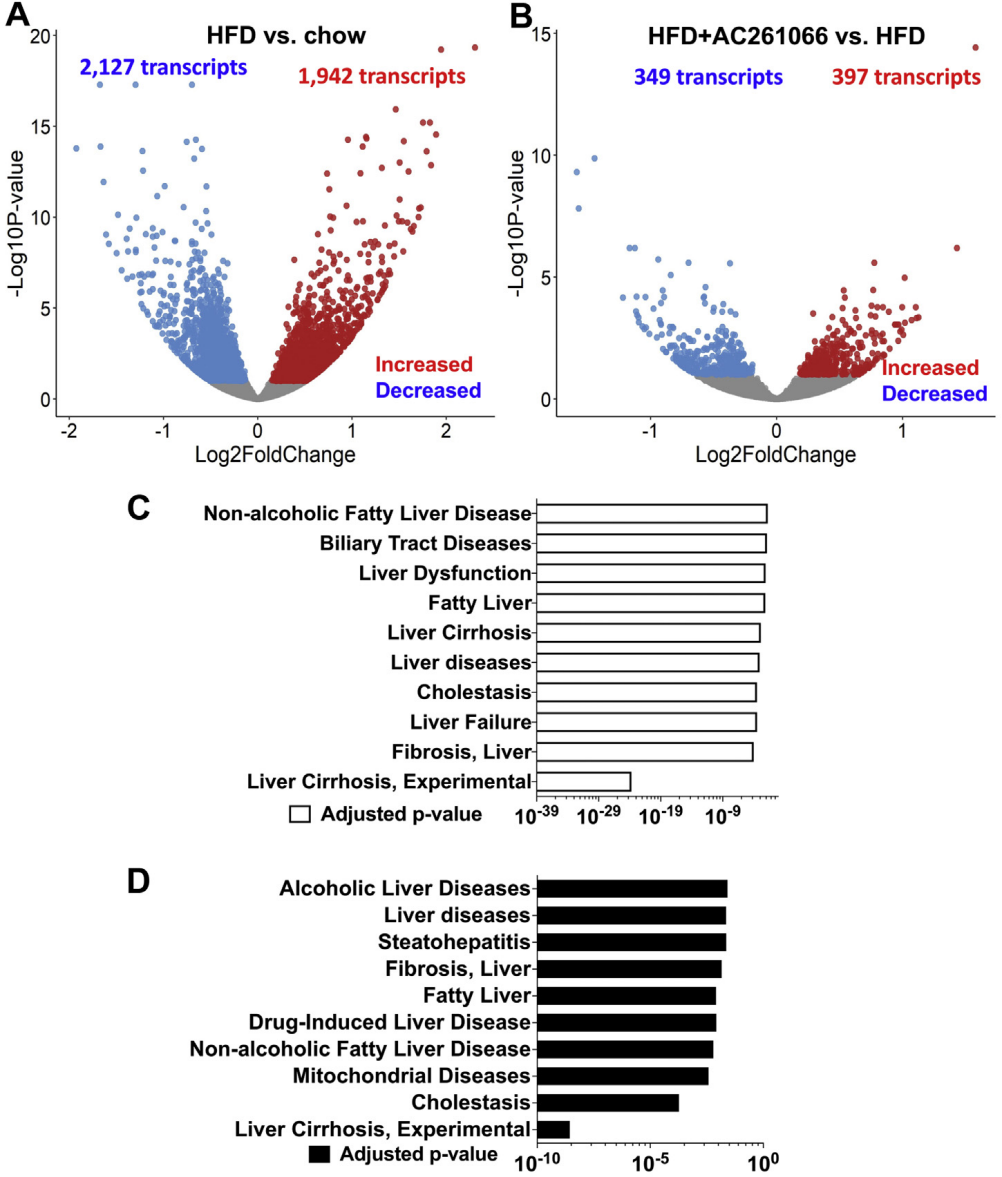
**Disease signature pathway analysis shows that AC261066 limits/prevents high-fat diet (HFD)-induced changes in the transcripts underlying development of NAFLD.***A*, volcano plot showing total numbers of transcripts with statistically significant increases or decreases (q < 0.1) in the HFD compared with the chow group (n = 6 per group). *B*, volcano plot showing total numbers of genes with statistically significant increases or decreases (q < 0.1) in the HFD+AC261066 compared with the HFD group (n = 6 per group). *C*, disease signature pathways increased in the HFD/chow. *D*, disease signature pathways decreased in the HFD+AC261066/HFD.

Here we found that AC261066 effectively limited liver steatosis and glucose excursion induced by a HFD with 60% kcal from fat, supporting our previous findings in a similar DIO (diet-induced obesity) model (14, 15, 16) (Fig. S1). Thus, we used this 60% HFD model to explore further the molecular mechanisms by which AC261066 exerts these potentially beneficial effects in the livers of these HFD-fed mice. Since RARβ is a transcription factor in the nuclear receptor superfamily (9), we next explored the mechanism(s) of action of AC261066 by using RNA-seq to assess global changes in liver transcripts. A total of 4069 transcripts (differentially expressed genes (DEGs)) differed significantly between the livers from HFD-fed and chow-fed mice, including increases in 1942 genes and decreases in 2127 transcripts, respectively (q < 0.1) (q = *p* value adjusted for the false discovery rate) (Fig. 1*A* and Table S1). Additionally, we found that compared with the HFD group, the HFD+AC261066 group showed 746 significantly changed DEGs, including increases in 397 transcripts and decreases in 349 transcripts (q < 0.1) (Fig. 1*B* and Table S1). Hierarchical clustering of DEGs in the different groups is shown in the heatmaps in Fig. S2 (*A*, HFD/chow; *B*, HFD+AC261066 (HFD+AC261)/HFD). Importantly, 225 transcripts increased in the HFD/chow were reduced in the HFD+AC261066/HFD, while 286 transcripts decreased in the HFD/chow were increased in the HFD+AC261066/HFD (Table S2). These gene overlaps between HFD/chow and HFD+AC261066/HFD were more significant than expected random chances, with a *p*-value < 2.2e-16 using Fisher’s exact tests. These data indicate that AC261066 limits the HFD-induced transcript changes in the liver.

To probe the relevance of our HFD model to human NAFLD, we used disease signature pathway analysis by DisGeNET (17) (Fig. 1, *C* and *D* and Table S3), as a discovery platform designed to probe a variety of gene and disease associations. The disease signature analysis revealed that the increased transcripts in the HFD/chow were associated with signature/pathways related to NAFLD, including “Liver cirrhosis experimental,” “Fibrosis, Liver,” “Fatty Liver,” “Cholestasis,” and “Nonalcoholic Fatty Disease” (Fig. 1*C*). The decreased transcripts in the HFD+AC261066/HFD were also associated with signature/pathways related to NAFLD, including “Liver cirrhosis experimental,” “Fibrosis, Liver,” “Fatty Liver,” “Cholestasis,” and “Nonalcoholic Fatty Disease” (Fig. 1*D*). These data indicate that our HFD mouse model is clinically relevant and mimics the gene signature of advanced human NAFLD, including liver fibrosis and liver cirrhosis, and that AC261066 impacted some pathways involved in NAFLD development.

Using the Gene Set Enrichment Analysis (GSEA) with Gene Ontology (GO) Biological Process Database (18), we discovered that compared with the chow group, the pathways with transcripts increased in the HFD group included “Arachidonic acid metabolic process” and “Long-chain fatty acid metabolic process,” while pathways with transcripts decreased in the HFD group included “Cytoplasmic translation” and “Cellular response to glucocorticoid stimulus.” Compared with the HFD group, the transcripts that decreased in the HFD+AC261066 group were enriched in “Extracellular matrix organization,” “Fatty acid metabolic process,” “Long-chain fatty acid metabolic process,” “Unsaturated fatty acid metabolic process,” and “Tricarboxylic acid metabolic process.” This suggests that these transcript changes were associated with HFD-induced NAFLD and AC261066’s protective actions on NAFLD (Figs. 1 and S1). Of note, we found that 282 transcripts changed in the HFD/chow are also altered by HFD in a previous study (19) (Table S4).

### AC261066 attenuates HFD-induced increases in the mRNA and protein levels of NAFLD driver genes

**Figure 2:**
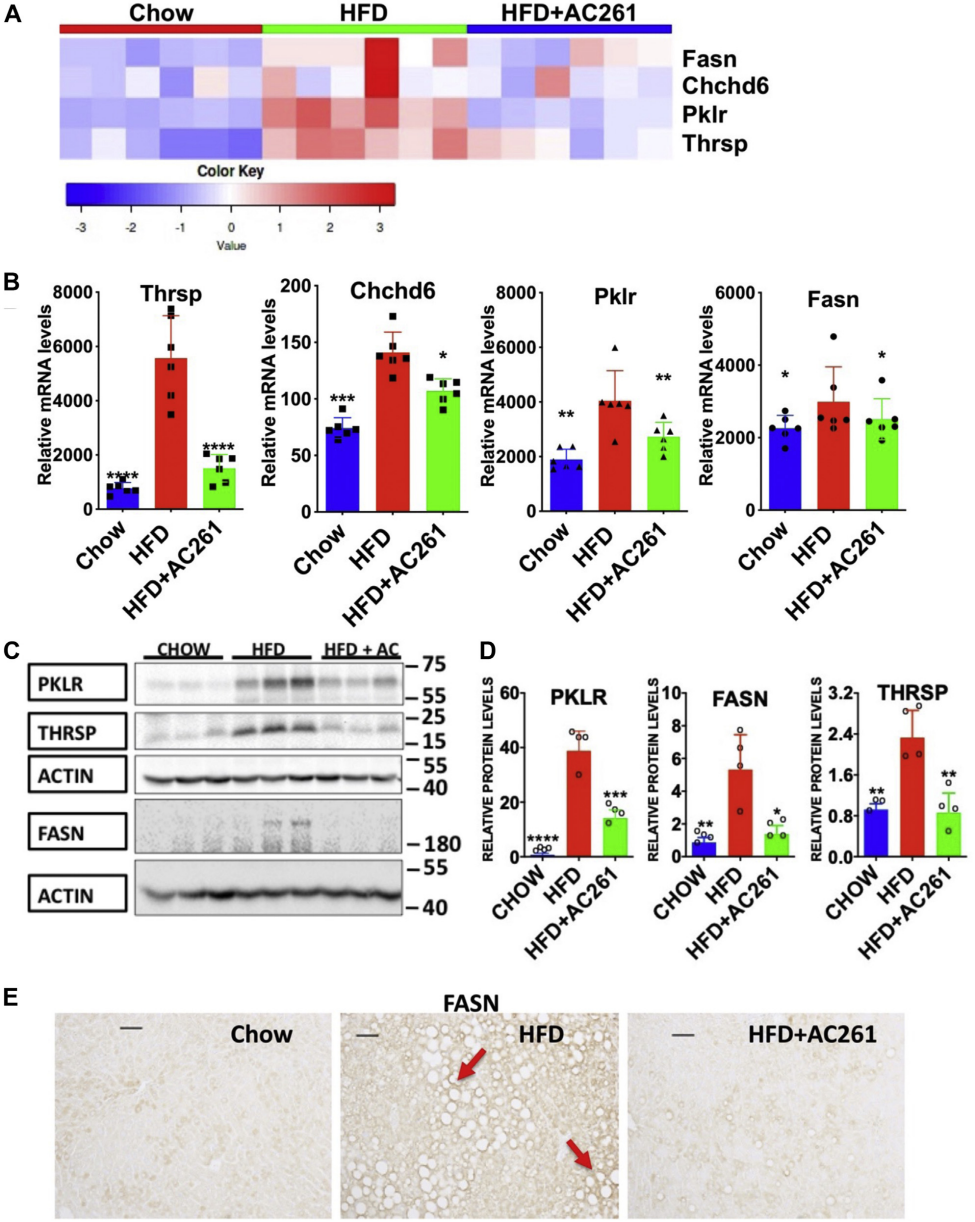
**Transcript and protein levels of NAFLD driver genes are increased in the HFD compared with the chow group, and AC261066 (AC261) ameliorates these changes.***A*, heatmap analyses of transcript levels of NAFLD driver genes from individual samples in all groups (n = 6 per group). *B*, the same data as in (*A*), but with statistical significance shown. Quantitative comparison of mRNA levels of the NAFLD diver genes from the RNA-seq data. The y axes (relative mRNA levels) are differentially expressed gene (DEG) transcript levels (n = 6 per group). *C*, immunoblotting of the hepatic protein levels of PKLR, FASN, and THRSP (Cropped images). Each lane is a sample from one mouse. Multiple Westerns were run to obtain quantitative data in (*D*). *D*, quantification of immunoblotting data (*C*). *E*, immunohistochemical analysis of FASN protein (*arrows*) in the liver sections from all groups. Scale bar = 50 μm. HFD+AC261=HFD+AC261066. ∗∗∗∗*p* < 0.0001, ∗∗∗*p* < 0.001, ∗∗*p* < 0.01, ∗*p* < 0.05, compared with the HFD group.

Using multiomics data, Krishnan *et al.* (20) reported key driver genes underlying NAFLD progression: *Pklr* (Pyruvate Kinase L/R), *Fasn*, *Thrsp*, and *Chchd6*. We also observed significantly greater hepatic mRNA levels of these four genes in the HFD group compared with the chow group (Fig. 2). Interestingly, these transcript levels were lower in the HFD+AC261066 (Abbreviation AC261) compared with the HFD group (Fig. 2, *A* and *B*). Further, the protein levels of these driver genes, PKLR, THRSP, and FASN, were greater in the HFD group by 51.8, 1.5, and 5.1-fold, respectively, than in chow. These proteins, especially PKLR and FASN, were lower by 2.7 and 3.9-fold, respectively, in the HFD+AC261066 compared with the HFD group (Fig. 2, *C* and *D*). Moreover, in the HFD group, FASN protein was detected in the livers enriched for lipid droplets (Fig. 2*E*). Taken together, these data suggest that AC261066 mitigates HFD-induced NAFLD, at least in part, by regulating NAFLD driver gene expression.

### AC261066 alters expression of genes contributing to lipogenesis in the liver

**Figure 3:**
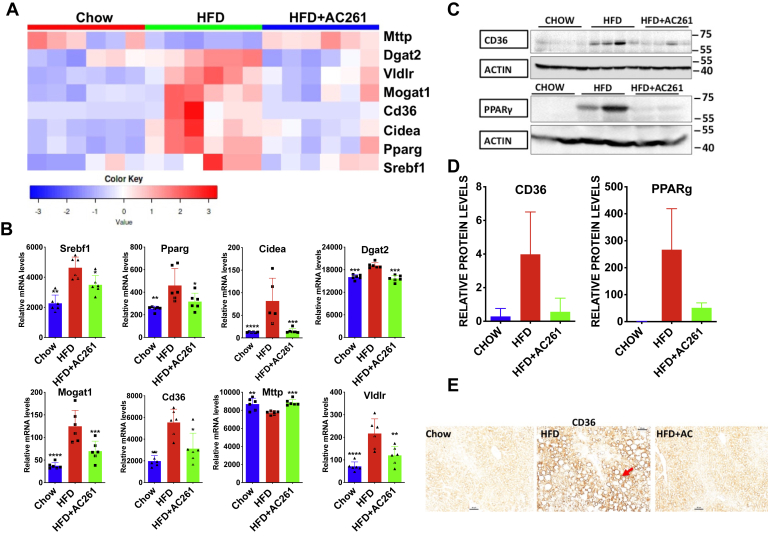
**HFD induced increases in the genes involved in hepatic lipogenesis and AC261066 (AC261) limits these increases.***A*, heatmap analyses of the transcript levels of genes involved in lipogenesis among all groups (n = 6 per group). *B*, the same data as in (*A*), but with statistical significance shown. Quantitative comparison of mRNA levels of lipogenesis related genes from the RNA-seq data. The y axes (relative mRNA levels) are differentially expressed gene (DEG) transcript levels (n = 6 per group). *C*, immunoblotting analysis of the hepatic protein levels of CD36 and PPARγ (Cropped images). Each lane is a sample from one mouse. *D*, quantification of the immunoblotting data from six mice/group (*C*). *E*, immunohistochemical analysis of CD36 protein (arrow) in the liver sections from all experimental groups. Magnification is 200×; scale bar is 50 μm. HFD+AC261=HFD+AC261066. ∗∗∗∗*p* < 0.0001, ∗∗∗*p* < 0.001, ∗∗*p* < 0.01, ∗*p* < 0.05, compared with the HFD group.

This HFD-driven NAFLD model produced severe liver steatosis (*i.e.*, >66% of fat within the hepatocytes (21), and AC261066 limited lipid accumulation in the liver (Fig. S1, *B* and *C*)). Therefore, we assessed the key genes involved in *de novo* lipogenesis, fatty acid import, and disposal in the liver. In line with our previous report (15), the heatmap and the quantitative comparison of the transcript levels derived from the RNA-seq data (Fig. 3, *A* and *B*) show that AC261066 mitigated the HFD-induced changes in key transcripts involved in these processes, including *Pparg* and *Srebf1*. AC261066 also greatly reduced the HFD-induced increase in PPARγ protein (Fig. 3, *C* and *D*). Transcript levels of *Scd1*, *Acaca* (ACC), and *Mlxipl* (ChREBP), genes involved in *de novo* lipogenesis, did not differ among all groups (Fig. S3).

CD36, a fatty acid translocase protein regulated by PPARγ, mediates uptake of circulating fatty acids by the liver and contributes to the increased uptake of lipids in NAFLD and NASH (22). *Cd36* mRNA levels in the HFD and HFD+AC261066 groups were 2.8-fold and 1.6-fold higher, respectively, than in chow (Fig. 3, *A* and *B*). Strikingly, the CD36 protein levels in the HFD and the HFD+AC261066 groups were 13.6- and 1.9-fold higher, respectively, than in the chow group (Fig. 3, *C* and *D*). In the HFD group, we detected the CD36 protein in liver parenchyma with the highest levels of lipid droplets, and CD36 was enriched on the plasma membrane (Fig. 3*E*). In contrast, we detected lower levels of CD36 in both the chow and the HFD+AC261066 groups, consistent with previous reports (22) (Fig. 3*E*). Thus, part of AC261066’s actions may be explained by reduced fatty acid uptake in the liver.

*Dgat2* and *Mogat1* play crucial roles in the synthesis of triglycerides, the primary storage form of intracellular lipid (23). *Cidea* mRNA, important for lipid droplet formation, is expressed at a low level in healthy liver and is robustly increased in steatosis (24). AC261066 attenuated HFD-induced increases in hepatic *Dgat2*, *Mogat1*, and *Cidea* mRNAs (Fig. 3*B*), suggesting that this may be another effector pathway for AC261066.

MTTP (microsomal triglyceride transfer protein) and VLDL (very low density lipoprotein) play primary roles in the export of triglycerides from the liver (25), and VLDLR (VLDL receptor) hinders the function of VLDL by binding to them. Compared with the chow group, HFD decreased *Mttp* and *Vldlr* mRNAs, respectively, and in the HFD+AC261066 group these changes were attenuated (Fig. 3, *A* and *B*). Collectively, these transcript data suggest that AC261066’s effects extend to multiple aspects of lipid metabolism, including endogenous lipogenesis and fatty acid transport.

### AC261066 attenuates HFD-induced changes in hepatic metabolite levels

To test whether these changes in gene expression are reflected at the metabolic level, we used an untargeted metabolomics approach (see Experimental procedures). We discovered that the liver levels of 343 metabolites were increased and 303 were decreased by HFD (*p* < 0.1). AC261066 increased 164 and decreased 172 metabolites (Table S5). Strikingly, the changes in some metabolites elicited by AC261066 in the HFD group were in opposite directions, as might be expected, suggesting that HFD alters hepatic metabolism and that AC261066 ameliorates some HFD-induced metabolite changes related to changes in the transcriptome and in the protein levels of key genes. In addition to the changes in triglyceride levels in the liver, shown in Fig. S1, metabolomics studies show that both HFD and AC261066 affect the levels of some fatty acids and other lipid species, including phosphatidylcholines (PC), phosphatidylethanolamine (PE), phosphatidylserine (PS), and lysophospholipids in the liver (Fig. S4). These changes in the phospholipids and lysophospholipids are quite interesting.

Pathway analysis shows that among the top pathways increased in the HFD/chow are “Warburg Effect,” “Fructose and Mannose Degradation,” “Ketone Body Metabolism,” “Glycolysis,” and “*De Novo* Triacylglycerol Biosynthesis”; among top pathways decreased in HFD/chow were “Lysine Degradation,” “Arginine/Proline Metabolism,” and “Tryptophan Metabolism” (Fig. S5*A*). In contrast, the top pathways decreased in the HFD+AC261066/HFD included “Warburg Effect” and “Fructose and Mannose Degradation,” whereas the top pathways increased included “Methionine Metabolism” and “Glycine/Serine Metabolism” (Fig. S5*B*). These data suggest that in our HFD-driven NAFLD model, some carbohydrate, lipid, and amino acid metabolism pathways are markedly altered as compared with the chow group, and that AC261066 treatment reversed/prevented these metabolite alterations.

### HFD increases fructose metabolism and AC261066 effectively attenuates this increase

**Figure 4:**
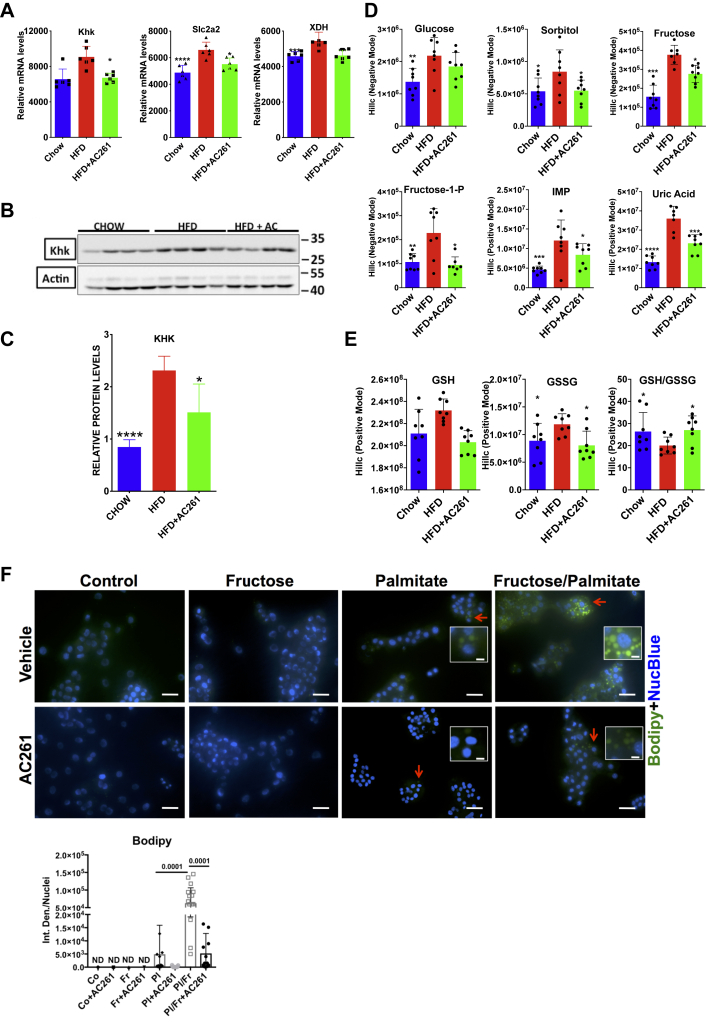
**HFD promotes fructose metabolism in the liver and AC261066 attenuates this effect.***A*, quantitative comparison of transcript levels of the genes involved in fructose uptake and metabolism from the RNA-seq data. The y axes (relative mRNA levels) are differentially expressed gene (DEG) transcript levels (n = 6 per group). *B*, immunoblotting analysis of the hepatic protein levels of KHK (Cropped images). Each lane is a sample from one mouse. *C*, quantification of the KHK immunoblotting data. *D*, the hepatic levels of metabolites involved in fructose metabolism in all experimental groups (n = 4 per group with two repeats). *E*, the hepatic levels of metabolites involved in oxidative stress in all experimental groups (n = 4 per group with two repeats). *F*, AML12 cells were stained with Bodipy reagent to label intracellular lipids after various treatments (Experimental procedures) for 48 h. Quantification of the staining with Fiji (ImageJ) representing the integrated density normalized/number of nuclei (NucBlue)/field. Representative areas highlighted in the *insets*. Scale bar for the images = 100 μm. Scale bar for the insets = 50 μm. HFD+AC261=HFD+AC261066. ∗∗∗∗*p* < 0.0001, ∗∗∗*p* < 0.001, ∗∗*p* < 0.01, ∗*p* < 0.05, compared with the HFD group. AC261, AC261066; Co, vehicle-treated cells; Fr, fructose; Pl, palmitate.

Fructose metabolism promotes *de novo* lipid biosynthesis in the liver and is hypothesized to be a key contributor to NAFLD progression, including NASH (26), and our data support this idea. Our RNA-seq data show that transcripts involved in fructose metabolism were increased in the HFD compared with the chow group, and these increases were attenuated in the HFD+AC261066 group, including (a) *Slc2a2* (Glucose transporter 2, GLUT2), a fructose transporter; (b) *Khk* (Ketohexokinase), the first fructose metabolizing enzyme that rapidly phosphorylates fructose to generate fructose-1-P (fructose-1-phosphate); and (c) *Xdh* (xanthine dehydrogenase), an enzyme that produces uric acid and contributes to oxidative stress (26) (Fig. 4*A*). The KHK protein levels also correlated with KHK mRNA levels: the HFD/chow ratio was 2.73 and the HFD+AC261066/chow ratio was 1.8 (Fig. 4, *A*–*C*). Additionally, *Khk* transcript levels in all treatment groups positively correlated with those of all NAFLD driver genes (Fig. S9*A*), the lipogenesis promoting transcription factors *Srebf1* and *Pparg*, and *Xdh* (Fig. S9*B*). These data suggest that fructose metabolism plays an important role in HFD-induced NAFLD development, and that AC261066 limits HFD-induced increases in fructose metabolism in the liver.

We also found that the ratios of fructose, fructose-1-P (fructose-1-phosphate), IMP (inosine monophosphate), and uric acid in HFD/chow were 2.4, 2.1, 2.6, and 2.7, respectively, indicating that fructose metabolism was increased by HFD. In the HFD+AC261066/chow, the ratios of fructose, fructose-1-P, IMP, and uric acid were 1.8, 0.9, 1.8, and 1.7, indicating that AC261066 limited these HFD-induced increases in fructose metabolites (Figs. S5*C* and 4*D*). Moreover, these metabolite levels were positively correlated with each other (Fig. S9*C*).

Since the dietary fructose in the chow and the HFD was comparable in our study (datasheets, Pico Diet, and BioServ Diet), we explored potential mechanisms related to the increase in the hepatic fructose level we observed in the HFD group. Endogenous fructose is produced from glucose through the sorbitol (polyol) pathway in which glucose is first converted to sorbitol by aldose reductase; then, sorbitol is further metabolized to fructose by sorbitol dehydrogenase. Hyperglycemia activates the polyol pathway (27). Although we did not detect changes in the mRNA levels of the enzymes aldose reductase (*Akr1b1*) and sorbitol dehydrogenase (*Sord*) in our RNA-seq data (Fig. S6), the hepatic sorbitol level in the HFD group was 1.6-fold greater than in chow, and the HFD+AC261066 group exhibited sorbitol levels comparable to those in the chow group (Fig. 4*D*). Fig. S9*C* shows a positive correlation between the levels of fructose and sorbitol. Therefore, the elevation of fructose in the HFD group likely resulted from both increased transport of fructose *via* GLUT2 and from the activated sorbitol pathway. Our data suggest that AC261066 negatively regulates both hepatic fructose transport and the hyperglycemia-activated polyol pathway.

To determine if these effects of AC261066 were direct effects on hepatocytes, we first employed the AML12 hepatocyte cell line. Neither control (vehicle-treated) or fructose-treated cells had detectable lipid droplets, while palmitate alone caused a low level of lipid droplet formation (Fig. 4*F*). Palmitate+fructose-treated cells exhibited a dramatic increase (>10-fold) in the lipid droplet level compared with other groups (Fig. 4*F*), indicating that fructose promotes endogenous lipogenesis. Importantly, we also found that AC261066 effectively limited the accumulation of lipid droplets induced by palmitate alone and the combination of palmitate and fructose, demonstrating that AC261066 can directly inhibit lipid accumulation in hepatocytes in this model system.

### RARβ is required for AC261066 to attenuate HFD-induced steatosis in hepatocytes

**Figure 5:**
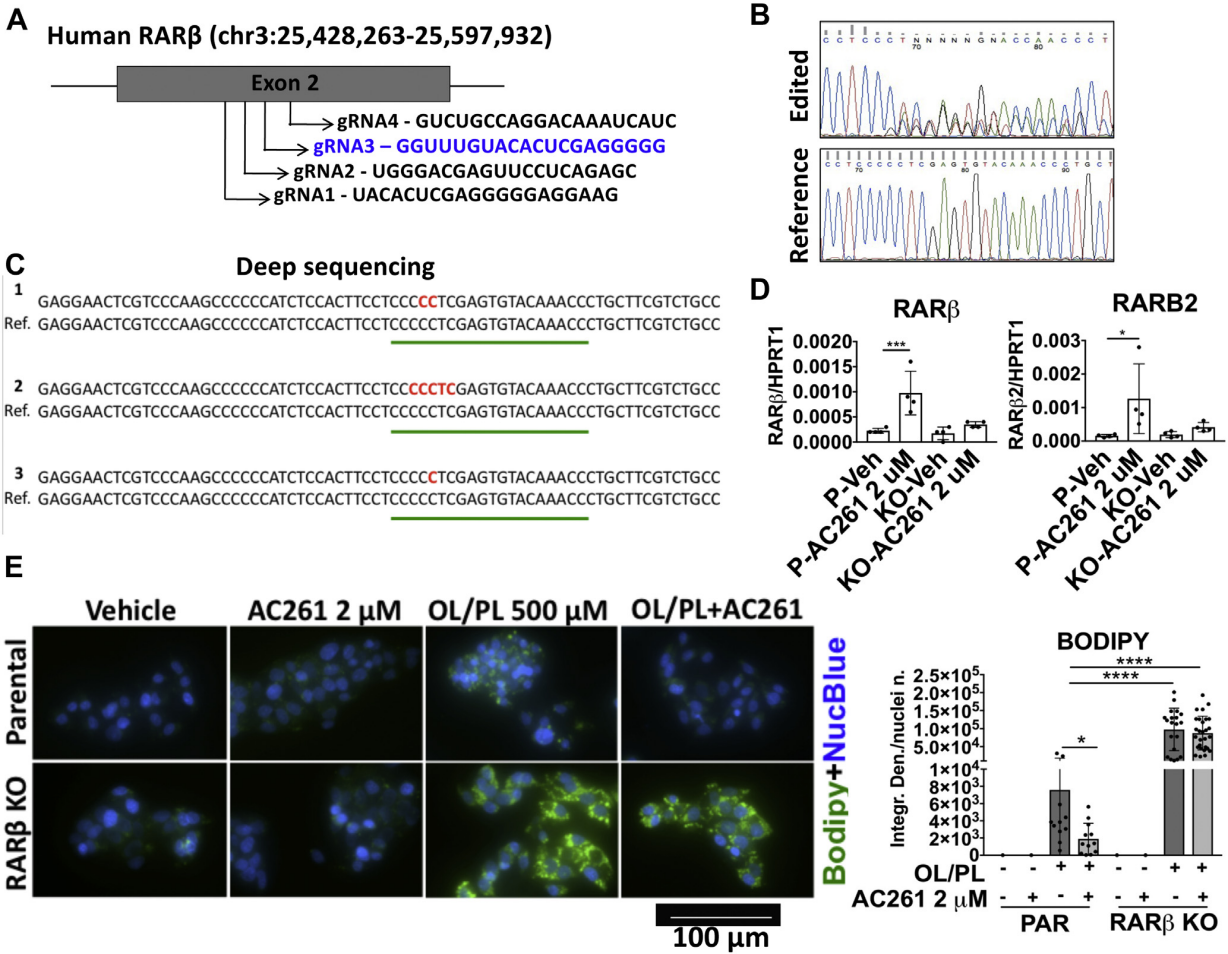
**AC261066 does not attenuate lipidaccumulation in RARβ knockout HepG2 cells.***A*, scheme of the Crispr/Cas9 technology strategy indicating the four guide RNAs (gRNAs) designed in exon 2 of the human RARβ gene. *B*, Sanger sequencing showing successful editing of one monoclonal colony identified in the gRNA highlighted in *blue*. *C*, next-generation sequencing (Deep Sequencing) performed to verify the mutations present in the Crispr/Cas9-edited cells. *D*, RARB and RARB2 mRNA levels measured in parental (P) and RARβ knockout (KO) HepG2 cells after treatment with 2 μM AC261066 for 72 h compared with vehicle-treated (veh) cells. Experiments were performed two times, each time in duplicate, with six wells/experimental group. *E*, oleate and palmitate (OL/PL; 2:1; 500 μM) treatment for 48 h in parental (PAR) and RARβ KO HepG2 cells in the presence or absence of AC261066 at a 2 μM concentration. The control groups are treated with the vehicle (0.1% DMSO). Bodipy staining lipid droplets in *green* and NucBlue staining nuclei in *blue*. Quantification of the staining with Fiji (ImageJ) representing the integrated density normalized/number of nuclei (NucBlue)/field of three independent experiments. ∗∗∗∗*p* < 0.0001, ∗∗∗*p* < 0.001, ∗∗*p* < 0.01, ∗*p* < 0.05, compared with the OL/PL-treated groups.

To elucidate whether AC261066, a selective RARβ2 agonist (13), limits liver steatosis *via* RARβ in hepatocytes, we examined AC261066’s effects on lipid accumulation in parental and RARβ knockout (RARβ KO) HepG2 cells generated using CRISPR/Cas9 technology (Fig. 5*A*). After determining the success of the Crispr/Cas9 editing by Sanger sequencing (Fig. 5*B*) and Next Generation Sequencing (Fig. 5*C*), we evaluated the effects of AC261066 on the mRNA levels of RARβ and RARβ2, the latter being the most abundant RARβ isoform. We found that in the HepG2 parental line after a 72 h treatment with 2 μM AC261066, the RARβ and RARβ2 mRNA levels were increased by 3.3-fold (±0.0004; *p* = 0.0007) and 7.3-fold (±0.001; *p* = 0.03), respectively (Fig. 5*D*). By contrast, we observed no changes in the HepG2 RARβ KO cells treated in parallel. Next, we treated parental and RARβ KO HepG2 cells with oleate and palmitate for 48 h ± 2 μM AC261066 and found that (i) RARβ KO HepG2 cells accumulated much greater levels of lipids (10.76-fold (±5.96; *p* < 0.0001)) than parental cells; (ii) treatment with AC261066 mitigated the accumulation of lipids in the parental cells by 3.39-fold (±5.5; *p* = 0.04) but did not show any effects in the oleate and palmitate+AC261066-treated RARβ KO cells (Fig. 5*E*). Thus, we show that RARβ plays a major role in regulating lipid accumulation in human hepatocytes in our *in vitro* culture model and that the mitigation of lipid accumulation by AC261066 treatment occurs *via* RARβ.

### AC261066 attenuates changes in redox potential caused by the HFD

Additionally, an appropriate GSH/GSSG (Glutathione/Glutathione disulfide) ratio is important in maintaining the normal cellular redox status in cells (28). We found that the GSH/GSSG ratio in HFD was 0.7-fold that in the chow group, and that the GSH/GSSG ratio in HFD+AC261066 was 1.4-fold that in HFD group (Figs. S5*C* and 4*E*), indicating that A261066 mitigated the HFD-induced alteration in cellular redox status.

### HFD induces changes in transcript levels of genes underlying NASH, and AC261066 limits these changes

Since fructose metabolism promotes NASH progression (26), we asked whether AC261066 affects markers of NASH. Indeed, 380 transcripts significantly altered in HFD/chow in our experiments are included in a gene signature of murine NASH (29) (Table S6). Additionally, 47 transcripts increased in this NASH gene signature (29) were reduced in HFD+AC261066/HFD; these include common markers of steatosis, such as *Srebf1*, *Pparg*, and *Cidea*, and transcripts involved in fibrogenesis, such as *Col1a1*, *Col1a2*, and *Mmp12* (14, 15, 29). Furthermore, 43 transcripts that showed decreases in the NASH gene signature (29) were increased in HFD+AC261066/HFD, including *Fgfr1*, *Foxo3*, and *Igfbp2* (Fig. S7 and Table S6). Taken together, these data indicate that AC261066 can mitigate the expression of genes involved in NASH progression.

### AC261066 limits the HFD-induced changes in transcripts within the hepatic stellate cells (HSC) that are associated with hepatocyte activation and fibrosis

**Figure 6:**
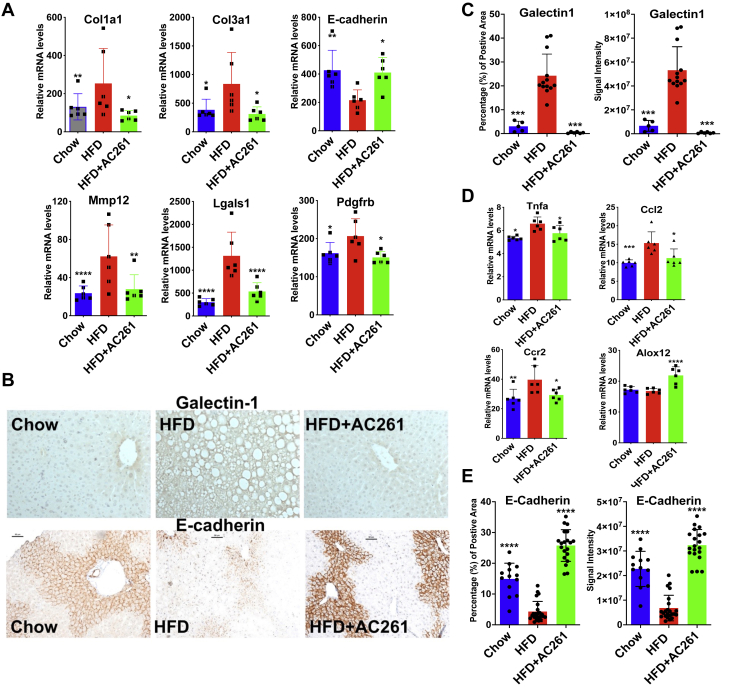
**HFD alters transcripts of genes involved in the hepatic stellate cell (HSC) secretome and AC261066 ameliorates these changes.***A*, quantitative comparison of representative transcripts contributing to liver fibrosis from RNA-seq data (n = 6 per group). *B*, immunohistochemical analysis of Galectin-1 and E-cadherin protein in liver sections from all experimental groups. Scale bar = 50 μm. *C* and *E*, quantification of images (*B*) on the signal intensity and the positively stained area using ImageJ. *D*, quantitative comparison of representative transcripts contributing to liver inflammation from the RNA-seq data (n = 6 per group). In (*A*) and (*D*), the y axes (relative mRNA levels) are differentially expressed gene (DEG) transcript levels. HFD+AC261=HFD+AC261066. ∗∗∗∗*p* < 0.0001, ∗∗∗*p* < 0.001, compared with the HFD group.

Single-cell RNA-seq studies (29) have identified a 128 gene secretome signature of HSCs, including secreted proteins and receptors. We previously reported that AC261066 mitigated HSC activation induced by HFD treatment (14). We compared transcripts significantly altered in HFD/chow and HFD+AC261066/HFD, respectively, with this 128 gene HSC secretome signature. Among these 128 secretome transcripts, 29 and 15, respectively, were altered in HFD/chow and/or HFD+AC261066/HFD (Fig. S8 and Table S6). The transcripts of many genes involved in fibrosis (30, 31), including *Col1a1*, *Col3a1*, *Lgals1* (Galectin-1), and PDGF receptor β (*Pdgfrb*), and the protein level of Galectin-1, were increased when comparing HFD with chow, and these increases were not as large when HFD+AC261066 was compared with HFD (Fig. 6, *A*–*C*). AC261066 also effectively modulated *Ccl2*, *Tnfa*, *Ccr2*, and *Alox12* transcripts involved in inflammation in NASH (Supporting information, Fig. 6*D*).

### AC261066 reverses the HFD-induced decrease in E-cadherin

E-cadherin is reduced in HFD models (32), the methionine–choline-deficient (MCD) model of NASH (33), and the CCl_4_ model of fibrogenesis (34). In our model, E-cadherin mRNA was markedly lower in the HFD than in the chow group, but this reduction was mitigated in the HFD+AC261066 group (Fig. 6*A*). E-cadherin protein was reduced by 3.5-fold in the livers from HFD-fed mice (Fig. 6*B*). The HFD+AC261066 mice actually showed higher E-cadherin protein levels than the chow group (Fig. 6, *B* and *E*). Thus, AC261066 limits HFD-induced changes in the expression of many liver-fibrosis-related genes.

## Discussion

**Figure 7:**
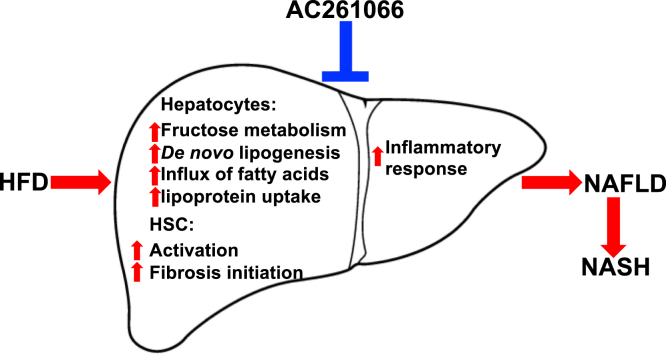
**Model of the effects of HFD and AC261066 in the liver**.

Analysis of changes in the transcriptome, protein levels of some key genes, and the metabolome in response to HFD compared with HFD plus AC261066 suggests that this RARβ2 agonist coordinates a cascade of events that leads to a reversal of many of the defining characteristics of NAFLD/NASH (Fig. 7). AC261066 attenuates changes in the driver genes that are thought to underlie the progression of this disease (20) (Fig. 2), and these changes in gene expression are accompanied by expected changes at the protein level and changes in metabolic intermediates. These changes lead to a reduction in the production and accumulation of triglycerides (Fig. S1). They offer an explanation for how fructose metabolism accelerates the progression of these disorders. We have also observed changes in the production of proteins that contribute to fibrosis and inflammation (Fig. 6), both of which are key pathological characteristics of NAFLD/NASH. Importantly, we provided direct evidence that AC261066 attenuates HFD-induced steatosis in hepatocytes *via* RARβ (Fig. 5). It is our plan to study the effects of AC261066 on other tissues using tissue/cell-specific RARβ knockout systems. We speculate that AC261066 acts directly on transcription and that this is the first step leading to a reduction in the HFD-associated pathological changes. We are also aware that changes in metabolites may directly or indirectly feed back on transcription and intermediary metabolism. This complexity does not negate the fact that AC261066 initiates a signaling pathway that reduces liver pathology, and we suggest that understanding the subsequent regulatory and signaling cascades involved will provide insights into the normal regulation of metabolism and how this regulation is changed by diet and drugs.

Retinoic acid mitigates hepatic stellate cell (HSC) activation and production of collagen I, III, and fibronectin that contribute to fibrosis (35). *In vitro* studies suggest that, through RARβ and RXRα, RA reduces type I collagen production in activated HSCs (36). In line with these studies, the changes in transcript levels of genes in the HSC secretome in the presence of AC261066 (Figs. 6 and S8) suggest that AC261066 could mitigate HSC activation and early fibrosis in mice *via* RARβ activation. Additionally, AM580, a RARα agonist, has a proinflammatory and profibrotic effect on lipopolysaccharide-activated HSCs (37), in agreement with our published study (16). Because different RARs have different and often antagonistic effects (38), our study emphasizes the potential of highly selective RARβ2 agonists for NAFLD/NASH treatment.

The primary source of CCL2 in the liver is monocytes or macrophages, and CCL2 expression is increased in Kupffer cells during NAFLD/NASH pathogenesis (39). Increases in CCL2 expression, an early event in NAFLD, lead to recruitment of the CCR2-expressing macrophages into the liver, which results in inflammation (40). In Kupffer cells, the *Rarb* transcript level is higher than that of *Rara* and *Rarg* (41). Thus, changes in *Ccl2* and *Ccr2* transcripts (Fig. 6) indicate that AC261066 could inhibit one of the early inflammatory responses involved in NAFLD/NASH development *via* RARβ in Kupffer cells.

Excessive fatty acids in the liver lead to toxic lipid species accumulation that induces liver stress and injury, resulting in fibrogenesis and inflammation, and targeting lipotoxicity inducing pathways is a focus in the rational design of therapeutic approaches for NASH (3). Retinoic acid suppresses *de novo* lipid synthesis and increases lipid oxidation in mouse liver (42), but in this earlier study the RARs involved were not elucidated. The two primary sources of free fatty acid flux that are crucial to the pathogenesis of NASH are free fatty acids delivered through blood to the liver and *de novo* lipogenesis (3). Here we demonstrate that AC261066 inhibits HFD-induced, free fatty acid flux *via* key genes involved in both endogenous lipogenesis and *via* the fatty acid transporter CD36 (Fig. 3).

Even without a dietary supplement of fructose, the HFD-induced increase in hepatic fructose metabolism can strongly promote *de novo* lipogenesis in animals and humans (26). Patients with NASH and mice supplemented with fructose show an increased hepatic KHK level, and lack of KHK expression in mice leads to improvement in NAFLD (26). Thus, inhibition of fructose metabolism is considered an attractive therapeutic approach for NAFLD/NASH (43), and a recent study provides preclinical evidence supporting KHK inhibition to improve NAFLD/NASH (44). Indeed, we show in AML12 cells that AC261066 directly suppresses fructose-promoted, endogenous lipogenesis in hepatocytes (Fig. 4*F*). Therefore, the attenuation of hepatic fructose metabolism by AC261066 (Fig. 4) indicates that inhibition of fructose metabolism could be one of the key mechanisms by which AC261066 limits NAFLD/NASH development associated with HFD treatment.

The constellation of changes initiated by AC261066 limit liver pathology seen with NAFLD/NASH, suggesting that this or related compounds may be a useful therapy for humans. Therapy for these diseases is an important unmet need, and AC261066 may have significant advantages over other therapies that are currently in development. For example, obeticholic acid (farnesoid X receptor (FXR) agonist), one of the potential therapies for NASH in clinical trials (3), almost completely depletes the liver of retinyl palmitate in animal models (45); this is a potential danger signal, as the liver is the major storage site in the body for vitamin A (46). AC261066 does not deplete the liver of vitamin A (16). One major challenge of NAFLD/NASH development is safety and efficacy during long-term administration. In our study, the effective administration period of AC261066 in mice was 2 months (3.5–5.5 month old), approximately equivalent to 6 to 7 years in humans (https://www.jax.org/research-and-faculty/research-labs/the-harrison-lab/gerontology/life-span-as-a-biomarker). Thus, the treatment period is equivalent to treatment in adolescents to young adults. Together with our previous studies (14, 15, 16), this suggests that AC261066 has a potential as a long-term treatment for NASH.

## Experimental procedures

### Mice, diets, and drug treatments

Wild-type (wt) male C57BL/6 mice (6–7 weeks old) were maintained on either a standard laboratory chow (Con) diet or a HFD with 60% kcal from fat for 4 months. Two months after the start of the HFD, HFD-treated mice were further maintained for 2 months in the absence or presence of 3 mg AC261066 per 100 ml drinking water (Supporting information).

### Glucose tolerance test (GTT)

We performed glucose tolerance testing as described (8, 15) (Supporting information). The care and use of animals in this study were approved by the Institutional Animal Care and Use Committee (IACUC) of Weill Cornell Medical College.

### Tissue dissection and pathological diagnosis

We dissected livers immediately after cervical dislocation after fasting overnight. We stained paraffin-embedded liver samples with hematoxylin and eosin (H&E) or Masson's Trichrome (Poly Scientific) for collagen deposition (Supporting information).

### Liver triglyceride measurements

We extracted lipids from snap-frozen liver samples using the Folch method (47) and measured triglycerides with a triglyceride reagent kit (Wako Diagnostics).

### Genome-wide RNA-seq analysis

Mouse liver samples stored in RNA later at sacrifice were subjected to RNA-seq analysis. We extracted RNA and subjected samples to Next-Generation Sequencing (RNA-seq) at the Genomics Resources Core Facility, Weill Cornell Medical College. We performed bioinformatics analyses using Tophat, Cufflink, and DESeq2 package software (Supporting information).

### Quantitative RT-PCR

We synthesized cDNA from 1 μg of total RNA using the RevertAid First Strand cDNA Synthesis Kit (Thermo, Inc). We performed quantitative RT-PCR (q-PCR) using SYBR Green PCR master mix on an Agilent Mx3000P Real Time PCR system (Agilent, Inc). We used the following human primer sequences: RARβ2 forward 5′-3′ GCTCCAGGAGAAAGCTCTCAAAG and reverse 5′-3′ ATTTGTCCTGGCAGACGAAGC; RARβ forward 5′-3′ ATGACAGCTGAGTTGGACGA and reverse 5′-3′ GTCAGCACTGGAATTCGTGG; HPRT forward 5′-3′ TGCTCGAGATGTGATGAAGG and reverse 5′-3′ TCCCCTGTTGACTGGTCATT.

### Immunohistochemical analysis

We stained paraffin-embedded liver tissue sections with various antibodies (Supporting information).

### Immunoblotting analysis

We homogenized snap-frozen liver samples and lysed cells in protein extraction buffer. Total protein lysates (30 μg) were used for immunoblotting (48). The signal intensity was measured using a quantitative gel imaging station (Chemi Doc, Bio Rad) within the linear range and analyzed using ImageJ software (Supporting information).

### Untargeted metabolomics

We extracted metabolites from snap-frozen liver samples and subjected them to untargeted metabolomics (Supporting information).

### Lipogenesis in cultured AML12 and HepG2 cells

We cultured AML12 (ATCC, CRL-2254) hepatocytes and the human hepatocellular carcinoma cell line, HepG2, (HB-8065, ATCC) to examine the effects of AC261066 on lipid accumulation (Supporting information).

### Crispr/Cas9 technology

To delete a portion of both alleles of the RARβ gene in HepG2 cells, we used Crispr/Cas9 technology and used the Synthego online tool (Synthego) to design guide RNA sequences. The detailed methods are in Supporting information.

### Statistical analyses

We performed statistical analyses by one-way analysis of variance and subsequently, the Bonferroni test or the Tukey test for multiple comparisons. Differences with a *p* < 0.05 (two-tailed test) were considered statistically significant.

## Data availability

### Data deposition

All data are contained within the manuscript, except the data deposited into a publicly accessible repository as follows: the RNA-seq data in this paper were deposited in the Gene Expression Omnibus (GEO) database, www.ncbi.nlm.nih.gov/geo (accession no. GSE165855), embargoed until publication.

The metabolomic data were deposited in Metabolomics Workbench, www.metabolomicsworkbench.org (accession no. ST001680), embargoed until publication.

## Supporting information

This article contains supporting information (15, 47, 49, 50, 51, 52, 53).

## Conflict of interest

Weill Cornell Medicine (WCM) has filed patents on intellectual property in this manuscript and these were licensed to Sveikatal, Inc. L. J. G. and X.-H. T. are founders and have financial interests in Sveikatal, Inc. M. M., A. R., C. L., J. J., T. Z., and S. S. G. report no conflicts of interest associated with this publication. This does not alter our adherence to policies on sharing data and materials.
