# Supplementary material for: A retinoic acid receptor β2 agonist attenuates transcriptome and metabolome changes underlying nonalcohol-associated fatty liver disease
Source: J Biol Chem. 2021 Oct 21;297(6):101331. doi: 10.1016/j.jbc.2021.101331 (PMC8626588; doi:10.1016/j.jbc.2021.101331)
Supplement: Supporting information and Figures S1–S9 [file mmc7.pdf]

Title: A Retinoic Acid Receptor β2 Agonist Attenuates Transcriptome and Metabolome Changes Underlying Non-Alcohol-Associated Fatty Liver Disease

the Transcriptome and Metabolome Underlying Non-Alcohol Associated Fatty Liver Disease

Xiao-Han Tang [1], Marta Melis [1], Changyuan Lu[1], Andrew Rappa [1], Tuo Zhang [2], Jose Jessurun [3], Steven S. Gross [1], and Lorraine J. Gudas [1*]

1. Department of Pharmacology, Weill Cornell Medical College of Cornell University, New York, NY 10065
2. Genomics Resources Core Facility, Weill Cornell Medical College of Cornell University, New York, NY 10065
3. Division of Anatomic Pathology, New York Presbyterian Hospital, Department of Pathology and Laboratory Medicine, Weill Cornell Medical College of Cornell University, New York, NY 10065

* To whom correspondence should be addressed: Lorraine J. Gudas, Ph.D., Department of Pharmacology, Weill Cornell Medical College of Cornell University, 1300 York Avenue, New York, NY, 10065; telephone: +1-212-746-6250; email: ljgudas@med.cornell.edu

Supporting Information

MATERIALS AND METHODS

Mice, diets, and drug treatments. Wild-type (wt) male C57BL/6 mice (6-7 weeks old) were maintained on either a standard laboratory chow (Con) diet with 0.23% fructose and 13 % kcal fat (diet no. 5053, Pico Diet, St. Louis, MO), or a high fat diet (HFD) with 60% kcal from fat and less than 0.13% fructose (diet no. F-3282, BioServ, Fleminngton, NJ) for 4 months. This high fat model is a well-established model for NAFLD (15). Two months after the start of the HFD, the mice were further split into three groups for 2 months to remain on (i) chow plus drinking water with 0.2 % DMSO; (ii) HFD plus drinking water containing 0.2 % DMSO; or (iii) HFD plus 3 mg AC261066 per 100 ml drinking water with 0.2 % DMSO. We previously tested the effects of AC261066 at the 3 mg/100 ml dose in chow-fed mice and found no histopathologic changes compared with chow-fed mice (47). Thus, we did not include a group of "chow plus AC261066". At the termination of the treatments, after fasting overnight mice were sacrificed by cervical dislocation and liver samples were harvested for analysis. We conducted the experiment twice, and the total numbers of mice in each group were: n=8 for chow; n=11 for HFD; and n=8 for HFD+AC261066. The care and use of animals in this study were approved by the Institutional Animal Care and Use Committee (IACUC) of Weill Cornell Medical College.

Glucose tolerance test (GTT). Mice were injected intraperitoneally with 25% glucose in PBS at the dose of 1.0 g/kg of body weight after fasting overnight. Blood glucose measurements were performed on tail vein blood at 15, 30, 45, 60, and 120 min post-injection using a FreeStyle Lite blood glucose monitoring system (Abbott Diabetes Care, Inc.).

Liver Histopathology. Paraffin embedded liver samples were stained with hematoxylin and eosin (H&E) and for collagen deposition using Masson's Trichrome Kit and Picro-Sirius Red (Poly Scientific, Bayshore, NY). Dr. Jose Jessurun (Department of Surgical Pathology at New York-Presbyterian Hospital/Weill Cornell Medical College), in a blinded manner, performed a complete histopathology evaluation on H&E and trichrome/Picro-Sirius Red stained liver samples and scored for evidence of steatosis, steatohepatitis and fibrosis according to the Brunt criteria (49). H&E-stained liver sections from all experimental groups were evaluated with eight histological features: Ductular reaction (score 0–3), portal and lobular inflammation (0- none, 1-mild, 2-moderate, 3-severe), presence or absence of atypical cells comprising the ductular reaction, degree of steatosis (0-none, 1<30%, 2>30 but <60%, 3- >60%), type of steatotic vacuoles (microvesicular or macro vesicular) and ballooning degeneration (0-none, 1-occasional, 2-more than occasional, 3-numerous cells). Degree of fibrosis was determined using both Scheuer scheme (0–4) and the Ishak fibrosis scores (0–6).

Liver Triglyceride Measurement. We extracted lipids from snap-frozen liver samples using the Folch method (50). Briefly, we homogenized the frozen liver samples and extracted total lipids using chloroform: methanol (2:1) followed by a partition using 0.9% NaCl. We resuspended the lipid samples in 0.5% (v/v) Triton X-100 solution in water after the organic phase solvents were

evaporated under nitrogen gas. Then we used a Triglycerides Reagent kit (Waco Diagnostics, Richmond, VA) to measure triglyceride levels according to the manufactures' protocol. The triglyceride levels were normalized to tissue weight (mg).

Lipogenesis in *in vitro* cultured AML12 and HepG2 cells. We cultured AML12 (ATCC, CRL-2254) mouse hepatocytes in DMEM:F12 1:1 medium (11320-033, Gibco) supplemented with 10% FBS (S10250, R&D Systems), 10 μg/ml insulin, 5.5 μg/ml transferrin, 5 ng/ml selenium (iTX mix 354352, Corning), 40 ng/ml dexamethasone (D4902, Sigma), and 7 ng/ml glucagon (G2044, Sigma). For the experiments we plated 20,000 cells per well in 24-well plates and serum-fasted the cells overnight before each experiment. We incubated the cells with palmitate (1 mM) with 1% BSA (fatty acid-free BSA, 126609, Millipore) in the medium at 37°C for 2 hours followed by fructose (20 mM) (51) and/or AC261066 (4.5 μM) treatments for 48 hours. All treatments were in triplicates. We performed three independent experiments. To detect lipid droplets, we fixed the cells in 4% paraformalehyde in PBS for 10 minutes at room temperature. After 2 washes in PBS we stained the cells with 100 nM BODIPY 493/503 (D3922, Invitrogen) and the nuclear dye NucBlue (R37605, Invitrogen) for 30 minutes. We then washed with PBS and acquired >10 fields per treatment group with a Nikon TE2000-E microscope.

We purchased the human hepatocellular carcinoma cell line HepG2 from the American Type Culture Collection (HB-8065, ATCC) and cultured these cells in Dulbecco's modified Eagle's medium (DMEM) supplemented with 10% fetal bovine serum (FBS). We plated 1,500 HepG2 cells (RARβ KO HepG2, described in the next section) per well in glass bottom 96-well plates and serum-fasted the cells overnight before each experiment. Next, we incubated oleate and palmitate (2:1 ratio, and a final concentration of 0.5 mM) with 1% BSA (fatty acid-free BSA, 126609, Millipore) in the medium at 37°C for 2 hours prior to treating the cells. We used 6 wells per experimental group and performed the experiments three times. The different treatments consisted of veichle +/- 2 μM AC261066 and oleate/palmitate (OL/PL) +/- 2 μM AC261066. To visualize the accumulation of lipids and the nuclei, we used the same BODIPY and NucBlue protocol as outlined above.

Crispr/Cas9 Technology.

To delete a portion of both alleles of the RARβ gene in HepG2 cells we used the Synthego online tool (Synthego; Redwood City, CA) to design guide RNA sequences. We selected four guide RNA sequences with the highest probability of RARβ gene disruption. All sequences were adjacent to or within exon 2 of the RARβ gene, as depicted in Figure 5A. We plated 50,000 cells in each 24-well plate and after 24h we treated the cells with the four guide RNA sequences (Figure 5A), following the manufacturer's instructions (Synthego). Briefly, after reconstitution of the guide RNA sequences with TE buffer (10 mM Tris-HCl, 1 mM EDTA, Ph 8) to a 100 μM stock concentration, for each well we prepared a mix including 6 μl of the guide RNA stock, 1.5 μl of the mRNA Cas9 nuclease (L-7206; TriLink Biotechnologies), 0.75 μl of mCherry (L-7203; TriLink Biotechnologies), 6 μl of Lipofectamine Messenger Max (LMRNA003; Invitrogen), and 200 μl of Optimem 1X (31985-070; Gibco). After mixing and incubating at room temperature for 5 minutes, we combined this

solution with 800 μl DMEM for a total of 1 mL and dispensed it in the wells. We then transfected the HepG2 cells for 24h, followed by trypsinization and plating in 6 well plates to allow the cells to proliferate until >70% confluency. Then we extracted genomic DNA for testing and expanded the remaining cells. We performed Sanger sequencing on the genomic DNA to assess the editing by visual comparison of the guide RNA sequences with one well of cells that remained untreated and was used as a reference genome. We also used the online tool provided by Synthego, Inference of CRISPR Edits (ICE), which revealed potential editing in cells treated with one of the four guide RNA (SI Appendix, Fig. 5) by providing a score indicative of the percentage of editing. The highest score resulting from this analysis was 15% in one guide RNA. We then performed single cell selection from this polyclonal population by plating 1 x 10^3 cells per 150 mm dish, and collected cells from 10 colonies after two weeks. We verified the success of CRISPR editing by Sanger sequencing by using the Synthego ICE tool, which showed a 96% editing efficiency. We also performed deep DNA sequencing of this HepG2-RARβ KO1 cell line and discovered three main frameshift mutations that should affect the function of the RARβ protein (Figure 5A, 5B).

Genome-Wide Transcriptomic Analysis

Liver total RNA preparation. We prepared total RNA from liver samples using the RNeasy kit (Qiagen). Subsequent steps were carried out at the Genomics Resources Core Facility of WCMC. RNA integrity was measured using the Agilent 2100 BioAnalyzer (Agilent Technologies).
cDNA library construction. Samples with RNA integrity number (RIN) values of >9 were used to construct cDNA libraries. cDNA synthesis, end-repair, and ligation to the Illumina indexed adapters were performed from the RNA samples by the TruSeq RNA protocol (Illumina). Libraries of 250-300 bp cDNA sizes were PCR-amplified using Phusion DNA polymerase. Following the removal of mRNA strands by RNaseH, first strand cDNAs were used as templates to produce double strand cDNAs. The overhangs resulting from fragmentation were repaired to blunt ends. An 'A' base was added to the 3' end of cDNAs and subsequently the cDNAs were ligated to Illumina paired end (PE) adaptors that have a single 'T' base overhang at their 3' end. The cDNA-adaptor libraries were purified and enriched by 15 cycles of PCR. The enriched libraries were hybridized to a flow cell and amplified, resulting in ultra-high density flow cells with millions of clusters, each containing about 1,000 copies of the templates. The double stranded cDNA-adaptors were denatured and converted into single strand DNA, and then the template cDNAs were amplified one more time isothermally to produce surface-bound colonies. The clonal DNA clusters were linearized, free 3' OH ends blocked, denatured, hybridized to sequencing primers.

cDNA library sequencing and data analysis.
The libraries were sequenced on the Illumina HiSeq 4000 with paired-end 51 bps and ~30 million paired reads per sample. The Sequencing-by-Synthesis process used reversible terminators and a DNA polymerase modified to accept reversible terminator nucleotides. After each synthesis cycle the fluorescence of clusters was imaged with high sensitivity. Then the sequencing images were analyzed in three steps, image analysis, base calling, and sequence analysis. Paired-end transcriptome sequencing reads were aligned to the reference mouse genome (UCSC mm9

assembly) using Tophat v2.0.11. Raw read counts were calculated using HTseq-count (52). DESeq2 v1.6.3 was used to perform differential expression analysis, principal component analyses and sample clustering. The removeBatchEffect function from the limma package v3.22.7 was used to remove batch effects introduced in processing samples from different batches. For differential expression analysis, pairwise comparisons between two or more groups using parametric tests where read-counts follow a negative binomial distribution with a gene-specific dispersion parameter. Corrected p-values were calculated based on the Benjamini-Hochberg method to be adjusted for multiple testing. The heatmaps for genes of interest were generated by R pheatmap package software.

Immunoblotting analysis. Mouse liver samples were homogenized, lysed in protein extraction buffer (0.125M Tris-HCl, pH 6.8, 2% SDS, 2.5% beta-mercaptoethanol) and denatured by boiling. Total protein lysates (30 μg) were resolved on sodium dodecyl sulfate–polyacrylamide gels and transferred to a nitrocellulose membrane (Cat# 162-0115; Bio-Rad). The following antibodies were used: mouse anti-ACTIN (cat# Mab1501, 1:10,000, Millipore), rabbit anti-FASN (cat# 3180, 1:1,000, Cell Signaling Technology), mouse anti-THRSP (cat# SCBT 137178, 1:100, Santa Cruz Biotechnology), rabbit anti-PKLR (cat# AB 171744, 1:500, Abcam), rabbit anti-CD36 (cat# ab 133625, 1:1,000, Abcam), rabbit anti-KHK (cat# ab154405, 1:1,000, Abcam), and rabbit anti-PPARγ (cat# 2435, 1:500, Cell Signaling Technology). Membranes were developed with enhanced chemiluminescence (Cat# 32106; ThermoScientific). Signals were subjected to densitometry using ImageJ software for quantification.

Immunohistochemical analysis. Paraffin-embedded sections, prepared at 7 μm thickness, from at least three mice per treatment group were deparaffinized and rehydrated in graded EtOH concentrations and distilled water. For antigen retrieval, slides were immersed in diluted (3:320) citrate-based antigen unmasking solution (Cat# H-3300; Vector Laboratories) and the slides were treated with 3% hydrogen peroxide prepared in methanol. After blocking with phosphate-buffered saline containing 10% goat serum (for rabbit primary antibodies), the sections were incubated with the following antibodies overnight at 4°C: rabbit anti-Galectin (cat# STJ 93201, 1:100, St. Johns Lab), rabbit anti-E-cadherin (cat# 33955, 1:200, Cell Signaling Technology), rabbit anti-FASN (cat# 3180, 1:200, Cell Signaling Technology, Danvers, MA), and rabbit anti-CD36 (cat# ab 133625, 1:200, Abcam). After incubation with the primary antibodies, the slides were treated with secondary antibodies supplied in the Mouse on SuperPicture HRP Polymer Conjugate (Cat# 87–8963; Life Technologies) kit. Antibody signals were visualized by peroxidase reaction using 3,3'-diaminobenzidine as a chromogen. As a negative control, tissue sections were incubated in the absence of primary antibody to ensure specificity of the primary antibody.
Six non-contiguous areas from the anterior and posterior portions of each section were photographed for analysis, and three sections from three different mice were measured. Signals were subjected to ImageJ software for quantification.

Liver metabolite analysis

Liver metabolite extraction. The snap frozen liver samples were incubated with 600 µL of 3 mM monobrombimane (MBB) in CH3OH:H2O (80:20) at -20°C for 2 h, followed by 1 h incubation at 0°C. Here, MBB was used to react with thiols and protect them for further oxidation. This initial incubation was followed by tissue disruption using stainless steel beads in a TissueLyser (Qiagen) and an additional 30 min at -20°C. Extracts were centrifuged for 15 min at 13000 rpm to pellet insoluble material and supernatants were transferred to clean tubes. This extraction was repeated two additional times and all three supernatants were dried in a speed-vac (Savant) and stored at −80 °C until analysis. For normalization of sample analyses, post-extracted tumor pellets were solubilized in 800 µL of 0.2 M aqueous NaOH at 95 °C for 60 min and the pellet protein was determined using the BioRad assay, relative to bovine serum albumin standards (0–1.5 mg/mL). For metabolite analysis, dried tumor extracts were reconstituted in CH3CN:H2O (70:30) containing 0.025% acetic acid at a relative protein concentration of 10 µg/µL and 3 µL solution was injected for LC/MS.

Q-TOF LC/MS Data Acquisition and Analysis. The LC system comprised a Cogent Diamond Hydride™ (ANP) column (2.1×150 mm, 3.5 µm particle size; Microsolv Technology Corp, Eatontown, NJ), a Zorbax SB-AQ (RP) column (2.1×100 mm, 1.8 µm particle size, Agilent Technologies, Santa Clara, CA), and a Model 1260 Rapid Resolution LC system consisting of a binary pump, on-line degasser, thermostated dual 54-well plate autosampler and a thermostated column compartment (Agilent Technologies, Santa Clara, CA). A pre-column replacement filter frit (0.5 µm, Upchurch Scientific, Oak Harbor, WA) and rapid resolution cartridge (Eclipse XDB-C8, Agilent technologies) were placed in front of the ANP and RP columns, respectively, to prevent column clogging. The LC flow was coupled to an Agilent model 6230 accurate mass time-of-flight (TOF) mass spectrometer, equipped with dual spray electrospray ionization (ESI) source. A separate isocratic pump was used deliver an internal reference mass solution (ions m/z 121.0509 and 922.0093) to the second ESI source for continuous mass calibration during sample analysis. LC parameters were set as follows: 3 µL injection volume, 0.4 mL/min mobile phase flow rate, 25°C column temperature and 4°C autosampler temperature. The mobile phase for ANP separation consisted of 6 mM EDTA and 0.025% acetic acid in isopropanol:H2O (50:50) (solvent A) and 6 mM EDTA and 5mM ammonium acetate in CH3CN: H2O (90:10) (solvent B). Gradient steps were applied as follows: 0–1 min, 99% B; 1–15 min, to 20% B; 15.1–29 min, 0% B; 29.01-37 min, 99% B. The mobile phase for RP separation consisted of 1 mM ammonium formate and 0.1% formic acid in H2O (solvent A) and 0.1% formic acid in CH3CN (solvent B). The gradient was as follows: 0–2 min, 1% B; 2– 20 min, to 50% B; 20–25 min, to 85% B; 25-30 min, to 99% B; 30.01-36 min, 1% B. Both positive and negative mass spectra were acquired in 2 GHz (extended dynamic range) mode with 1.41 spectra/sec sampled over a mass/charge range of 40–1400 Daltons. The TOF capillary voltage was set at 4000 V for positive ions and 3500 V for negative ions with the fragmentor set to 175 V. The nebulizer pressure was 35 psi and the nitrogen drying gas was delivered at a flow rate of 12 L/min. Data was saved in both centroid and profile mode using Agilent MassHunter Workstation B500 Data acquisition Software. To minimize potential salt and other contaminants in the ESI source, a time segment was set for both ANP and RP positive and negative acquisitions that directed the first 0.2 mL of column elute to waste.

Raw data files were processed using MassHunter Qualitative Analysis Software (B06.00; Agilent Technologies). Downstream comparative data analysis was performed using MassProfiler Professional (Agilent, B14.5) and MassHunter Profinder (B07.00). The molecular feature extraction (MFE) searches compounds based on the profile of identical m/z values and retention times, within a defined mass accuracy (<5 ppm). These features are further grouped into one or more "compounds" based on their isotope pattern, the formation of dimer, adduct ions (e.g. H^+, Na^+, NH_4^+ for positive mode and H^-, CH_3COO^-, $HCOO^-$ and Cl^- for negative mode) and common neutral losses of H_2O and NH_3. The identified features were manually validated following extraction. The identification is further confirmed by comparison to pure chemical standards.

Untargeted metabolite profiling. As previously described (53), untargeted metabolite profiling was performed using a platform of an Agilent Model 1290 Infinity II liquid chromatography system coupled to an Agilent 6550 iFunnel time-of-flight MS analyzer. A Cogent Diamond Hydride™ (ANP) column and a Zorbax SB-AQ (RP) column were used for metabolites separation. The mobile phase for ANP separation consisted of 6 mM EDTA and 0.025% acetic acid in isopropanol: H_2O (50:50) (solvent A) and 6 mM EDTA and 5mM ammonium acetate in CH_3CN: H_2O (90:10) (solvent B). Gradient steps were applied as follows: 0–1 min, 99% B; 1–15 min, to 20% B; 15.1–29 min, 0% B; 29.01-35 min, 99% B. The mobile phase for RP separation consisted of 1 mM ammonium formate and 0.1% formic acid in H_2O (solvent A) and 0.1% formic acid in CH_3CN (solvent B). The gradient was as follows: 0–2 min, 1% B; 2– 20 min, to 50% B; 20–25 min, to 85% B; 25-30 min, to 99% B; 30.01-36 min, 1% B.

Raw data files were processed using MassHunter Qualitative Analysis Software (B07.00; Agilent Technologies). Downstream comparative data analysis was performed using MassHunter Profinder (B08.00) and MassProfiler Professional (Agilent, B14.5). The identified features were manually validated following extraction. The identification was further confirmed by comparison to pure chemical standards. Mann Whitney t-tests ($p < 0.05$) were performed to identify significant differences between groups. The results of glucose, fructose, fructose-1-P, fructose-6-P, and glucose-6-P were further confirmed with a Shodex HILICpak VT-50 2D column (2.0mmI.D. 150mm, Japan) using 25mM $HCOONH_4$ (H_2O): CH_3CN (80:20) as mobile phase.

REFERENCES

15. Recena Aydos, L., Aparecida do Amaral, L., Serafim de Souza, R., Jacobowski, A. C., Freitas Dos Santos, E., and Rodrigues Macedo, M. L. (2019) Nonalcoholic Fatty Liver Disease Induced by High-Fat Diet in C57bl/6 Models. Nutrients 11

47. Trasino, S. E., Tang, X. H., Jessurun, J., and Gudas, L. J. (2016) Retinoic acid receptor β2 agonists restore glycaemic control in diabetes and reduce steatosis. Diabetes Obes Metab 18, 142-151

49 Brunt, E. M., Kleiner, D. E., Wilson, L. A., Belt, P., Neuschwander-Tetri, B. A., and (CRN), N. C. R. N. (2011) Nonalcoholic fatty liver disease (NAFLD) activity score and the histopathologic diagnosis in NAFLD: distinct clinicopathologic meanings. Hepatology 53, 810-820

50. Folch, J., Lees, M., and Sloane Stanley, G. H. (1957) A simple method for the isolation and purification of total lipides from animal tissues. J Biol Chem 226, 497-509

51. Piras, I. S., Gerhard, G. S., and DiStefano, J. K. (2020) Palmitate and Fructose Interact to Induce Human Hepatocytes to Produce Pro-Fibrotic Transcriptional Responses in Hepatic Stellate Cells Exposed to Conditioned Media. Cell Physiol Biochem 54, 1068-1082

52. Anders, S., Pyl, P. T., and Huber, W. (2015) HTSeq--a Python framework to work with high-throughput sequencing data. Bioinformatics 31, 166-169

53. Goncalves, M. D., Lu, C., Tutnauer, J., Hartman, T. E., Hwang, S. K., Murphy, C. J., Pauli, C., Morris, R., Taylor, S., Bosch, K., Yang, S., Wang, Y., Van Riper, J., Lekaye, H. C., Roper, J., Kim, Y., Chen, Q., Gross, S. S., Rhee, K. Y., Cantley, L. C., and Yun, J. (2019) High-fructose corn syrup enhances intestinal tumor growth in mice. Science 363, 1345-1349

Figure S1

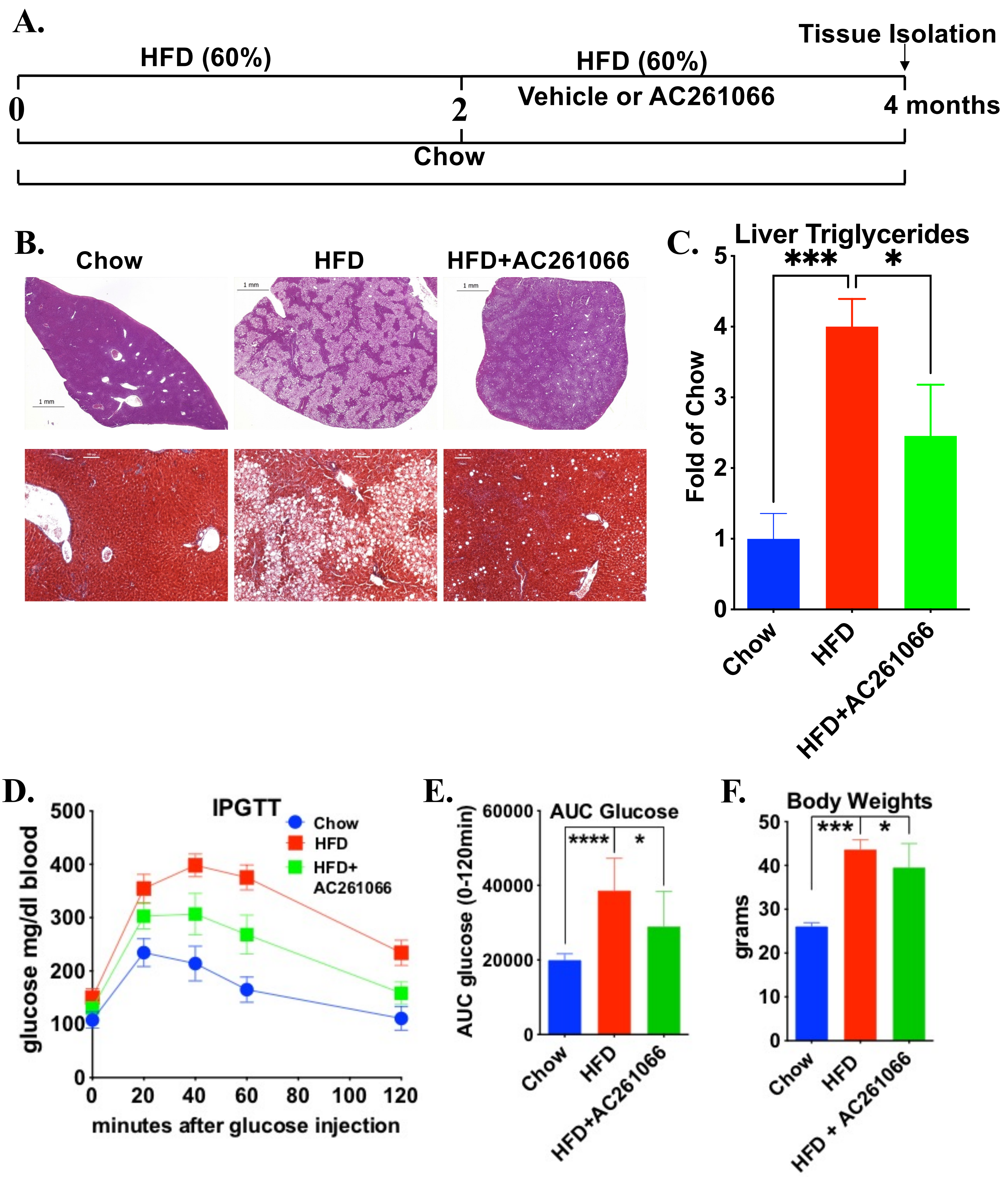
A.
Tissue Isolation
HFD (60%)
HFD (60%)
Vehicle or AC261066
0
2
4 months
Chow
B.
Chow
HFD
HFD+AC261066
C.
Liver Triglycerides
Fold of Chow
***
*
Chow
HFD
HFD+AC261066
D.
IPGTT
glucose mg/dl blood
minutes after glucose injection
Chow
HFD
HFD+
AC261066
E.
AUC Glucose
AUC glucose (0-120min)
****
*
Chow
HFD
HFD+AC261066
F.
Body Weights
grams
***
*
Chow
HFD
HFD + AC261066

Figure S1. AC261066, a retinoic acid receptor (RAR) beta 2 agonist limits high fat diet (HFD) induced liver steatosis and glucose intolerance in mice. (A) Diagram of the experimental protocol (Materials and Methods). **(B)** Representative gross morphology of the H&E-stained liver sections (top) (16x, scale bar is 1 mm), and representative trichrome stained liver sections (bottom) in this study (200x, scale bar is 50 μm). White vacuoles indicate lipid accumulation. **(C)** Liver triglycerides (mg/g of tissue) from mice described in **(A)**. AC261066 treatments effectively limited HFD-induced liver steatosis (Figure S1B) and increase in hepatic triglyceride level (Figure S1C). Eight weeks of AC261066 treatment greatly attenuated glucose excursion and the area under the curve (AUC) for glucose in this 60% HFD model (Figure S1D, E). Compared to chow-fed mice, 4 months of HFD feeding increased body weights (BW), and AC261066 had a modest effect on lowering body weight (Figure S1F).****$p < 0.0001$, *$p < 0.05$, compared to the HFD group.

Figure S2

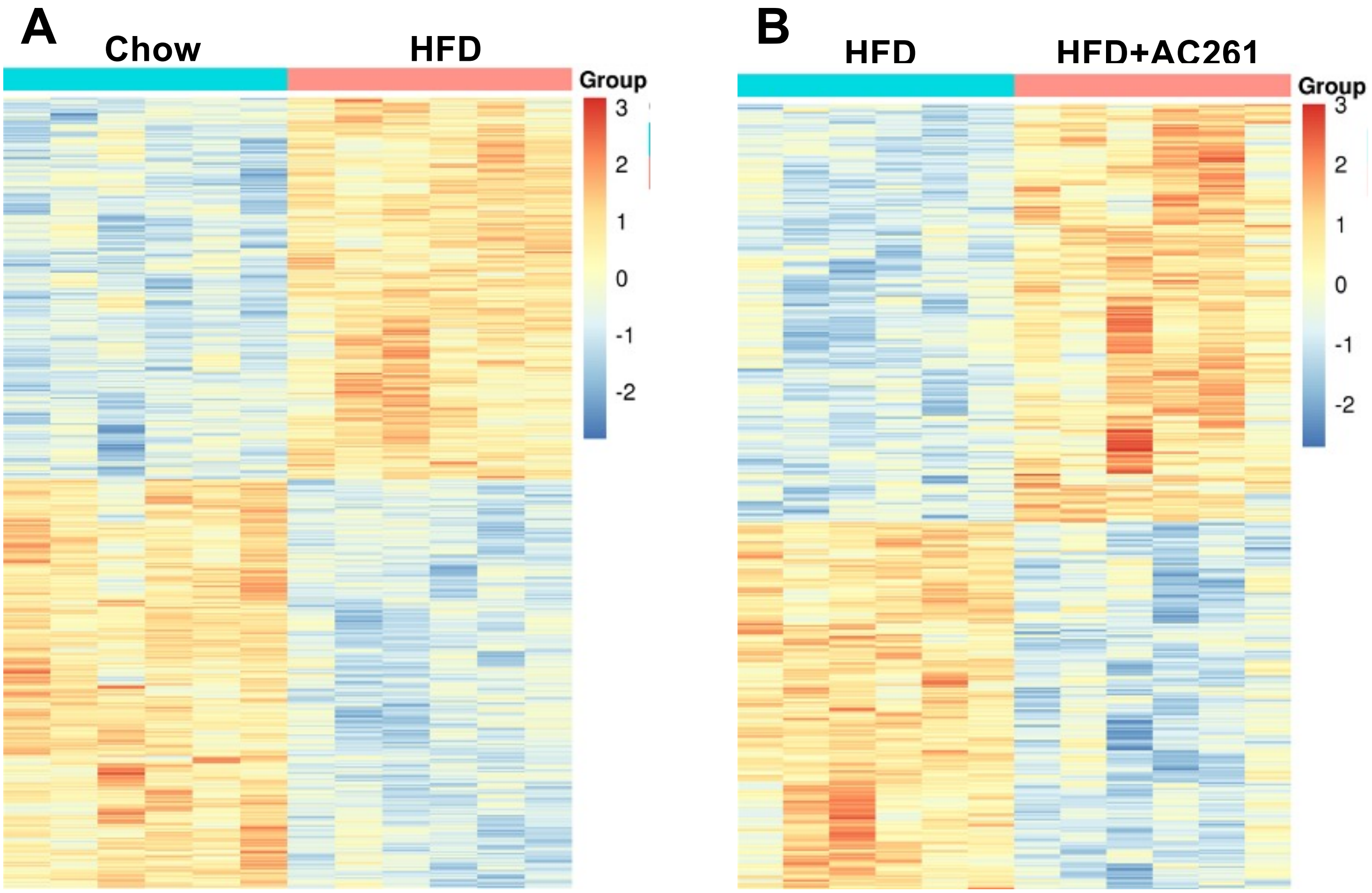

Figure S2. Global views of alterations in transcript levels by RNA-seq. The mRNA samples extracted from the liver were subjected to RNA-seq analysis. (A) Heatmap showing the total genes altered with statistical significance (q<0.1) in the HFD group compared to the chow group (n=6 per group). **(B)** Heatmap showing the total genes altered with statistically significance (q<0.1) in the HFD+AC261066 group, compared with the HFD group (n=6 per group). HFD+AC261=HFD+AC261066.

Figure S3

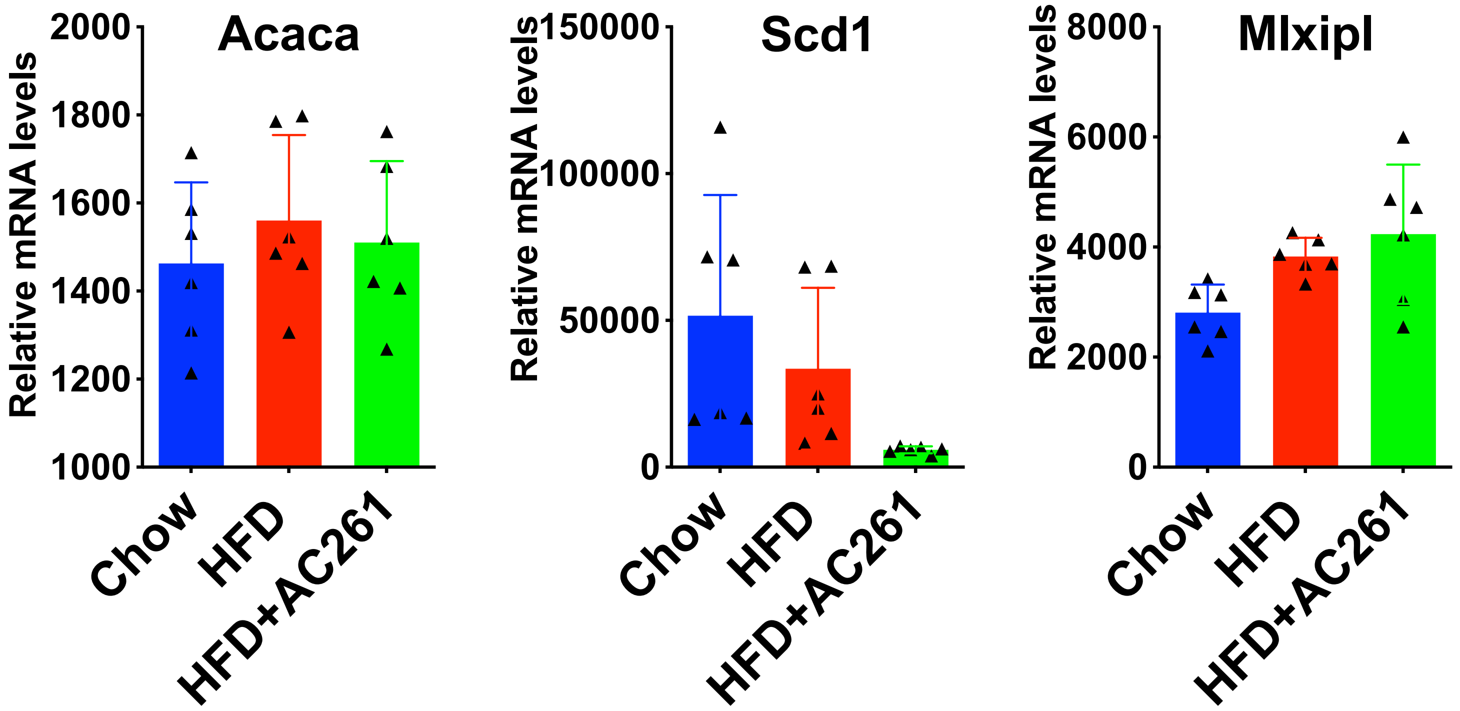
Acaca
Relative mRNA levels
2000
1800
1600
1400
1200
1000
Chow
HFD
HFD+AC261
Scd1
Relative mRNA levels
150000
100000
50000
0
Chow
HFD
HFD+AC261
Mlxipl
Relative mRNA levels
8000
6000
4000
2000
0
Chow
HFD
HFD+AC261

Figure S3. Comparison of mRNA levels of *Scd1*, *Acaca* (ACC), and *Mlxipl* (ChREBP) from the RNA-seq data (n=6 per group)**.** The y axes (relative mRNA levels) are differentially expressed gene (DEG) transcript levels. HFD+AC261=HFD+AC261066.

Figure S4

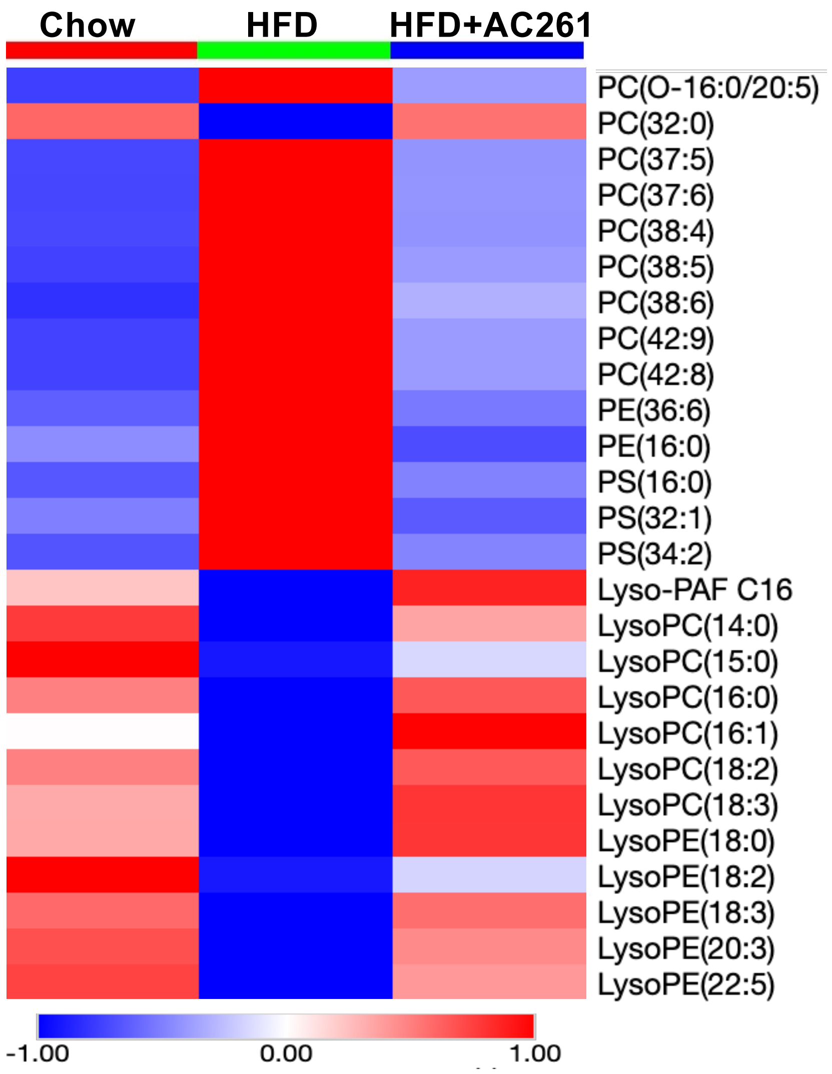
Chow
HFD
HFD+AC261
PC(O-16:0/20:5)
PC(32:0)
PC(37:5)
PC(37:6)
PC(38:4)
PC(38:5)
PC(38:6)
PC(42:9)
PC(42:8)
PE(36:6)
PE(16:0)
PS(16:0)
PS(32:1)
PS(34:2)
Lyso-PAF C16
LysoPC(14:0)
LysoPC(15:0)
LysoPC(16:0)
LysoPC(16:1)
LysoPC(18:2)
LysoPC(18:3)
LysoPE(18:0)
LysoPE(18:2)
LysoPE(18:3)
LysoPE(20:3)
LysoPE(22:5)
-1.00
0.00
1.00

Figure S4. Alterations in hepatic phospholipids and lysophospholipids levels measured by untargeted metabolomics. Heatmap showing the levels of phospholipids and lysophospholipids altered statistically in the HFD/chow and the HFD+AC261066/HFD. HFD+AC261=HFD+AC261066 (n=4 per group with 2 repeats).

Figure S5A

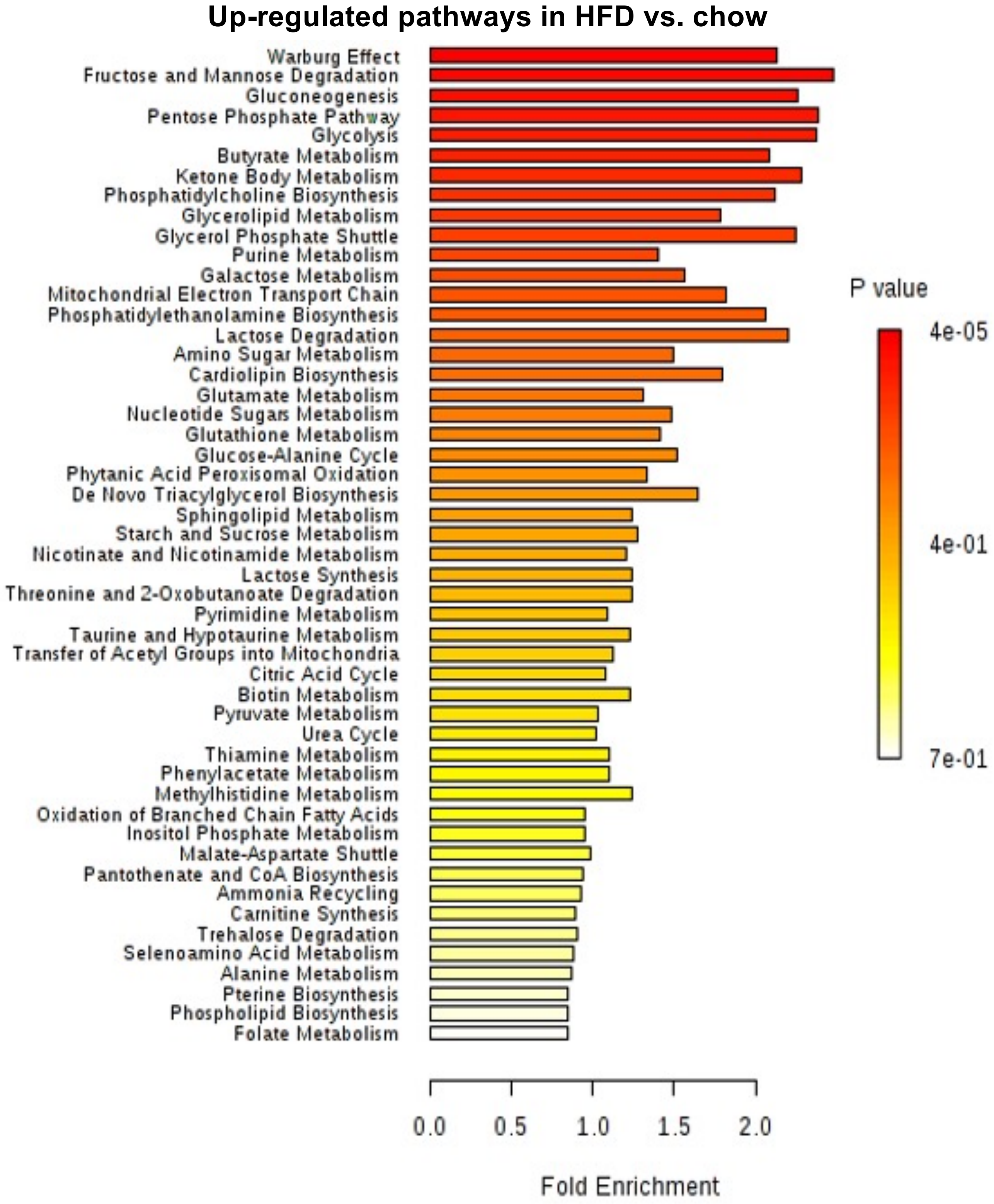
Up-regulated pathways in HFD vs. chow
Warburg Effect
Fructose and Mannose Degradation
Gluconeogenesis
Pentose Phosphate Pathway
Glycolysis
Butyrate Metabolism
Ketone Body Metabolism
Phosphatidylcholine Biosynthesis
Glycerolipid Metabolism
Glycerol Phosphate Shuttle
Purine Metabolism
Galactose Metabolism
Mitochondrial Electron Transport Chain
Phosphatidylethanolamine Biosynthesis
Lactose Degradation
Amino Sugar Metabolism
Cardiolipin Biosynthesis
Glutamate Metabolism
Nucleotide Sugars Metabolism
Glutathione Metabolism
Glucose-Alanine Cycle
Phytanic Acid Peroxisomal Oxidation
De Novo Triacylglycerol Biosynthesis
Sphingolipid Metabolism
Starch and Sucrose Metabolism
Nicotinate and Nicotinamide Metabolism
Lactose Synthesis
Threonine and 2-Oxobutanoate Degradation
Pyrimidine Metabolism
Taurine and Hypotaurine Metabolism
Transfer of Acetyl Groups into Mitochondria
Citric Acid Cycle
Biotin Metabolism
Pyruvate Metabolism
Urea Cycle
Thiamine Metabolism
Phenylacetate Metabolism
Methylhistidine Metabolism
Oxidation of Branched Chain Fatty Acids
Inositol Phosphate Metabolism
Malate-Aspartate Shuttle
Pantothenate and CoA Biosynthesis
Ammonia Recycling
Carnitine Synthesis
Trehalose Degradation
Selenoamino Acid Metabolism
Alanine Metabolism
Pterine Biosynthesis
Phospholipid Biosynthesis
Folate Metabolism
P value
4e-05
4e-01
7e-01
0.0
0.5
1.0
1.5
2.0
Fold Enrichment

Figure S5A, Continued

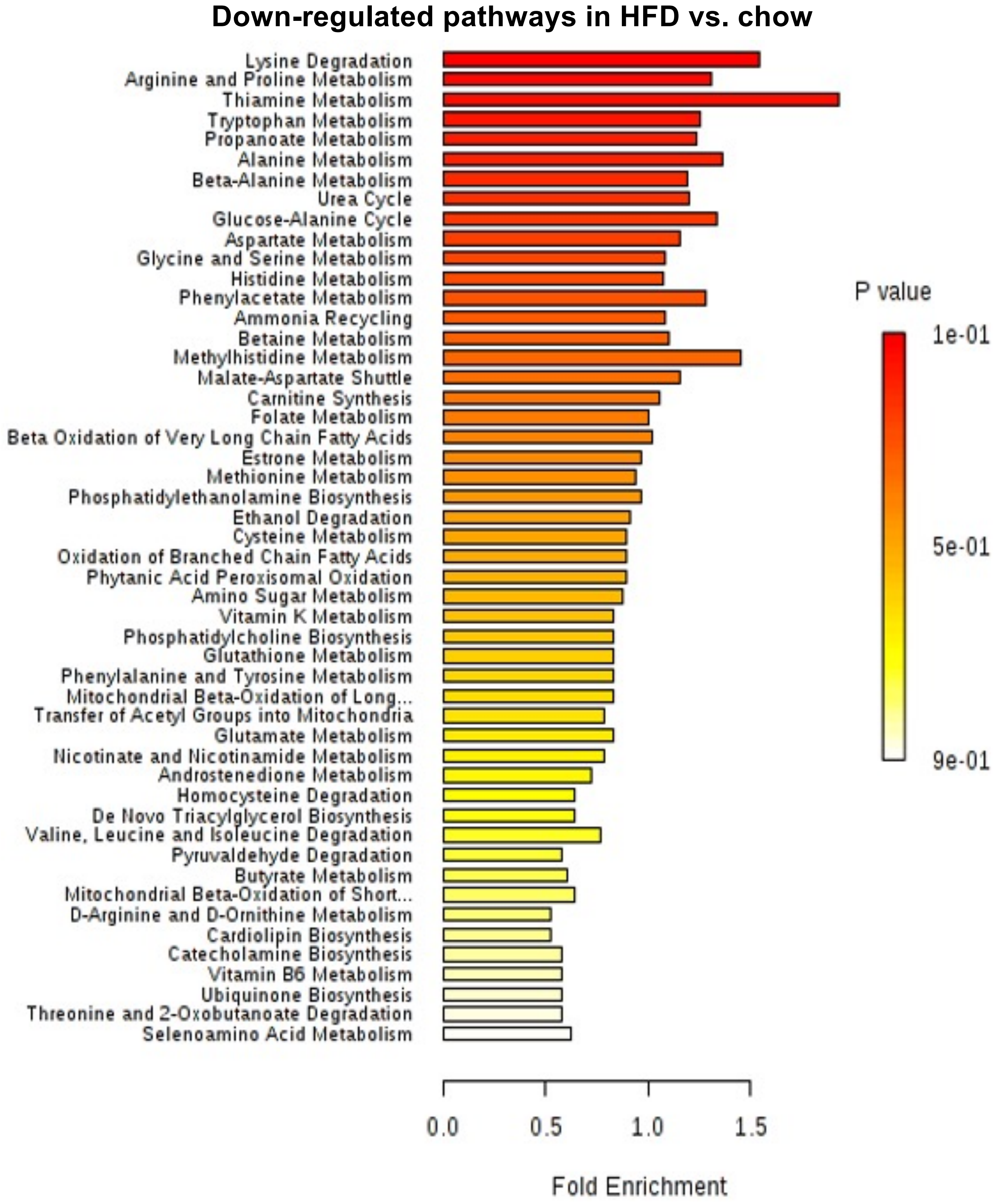

Figure S5A Alterations in pathways from hepatic metabolite levels measured by untargeted metabolomics. Pathways derived from the hepatic metabolites whose levels were significantly altered in the HFD/chow (n=4 per group with 2 repeats).

Figure S5B

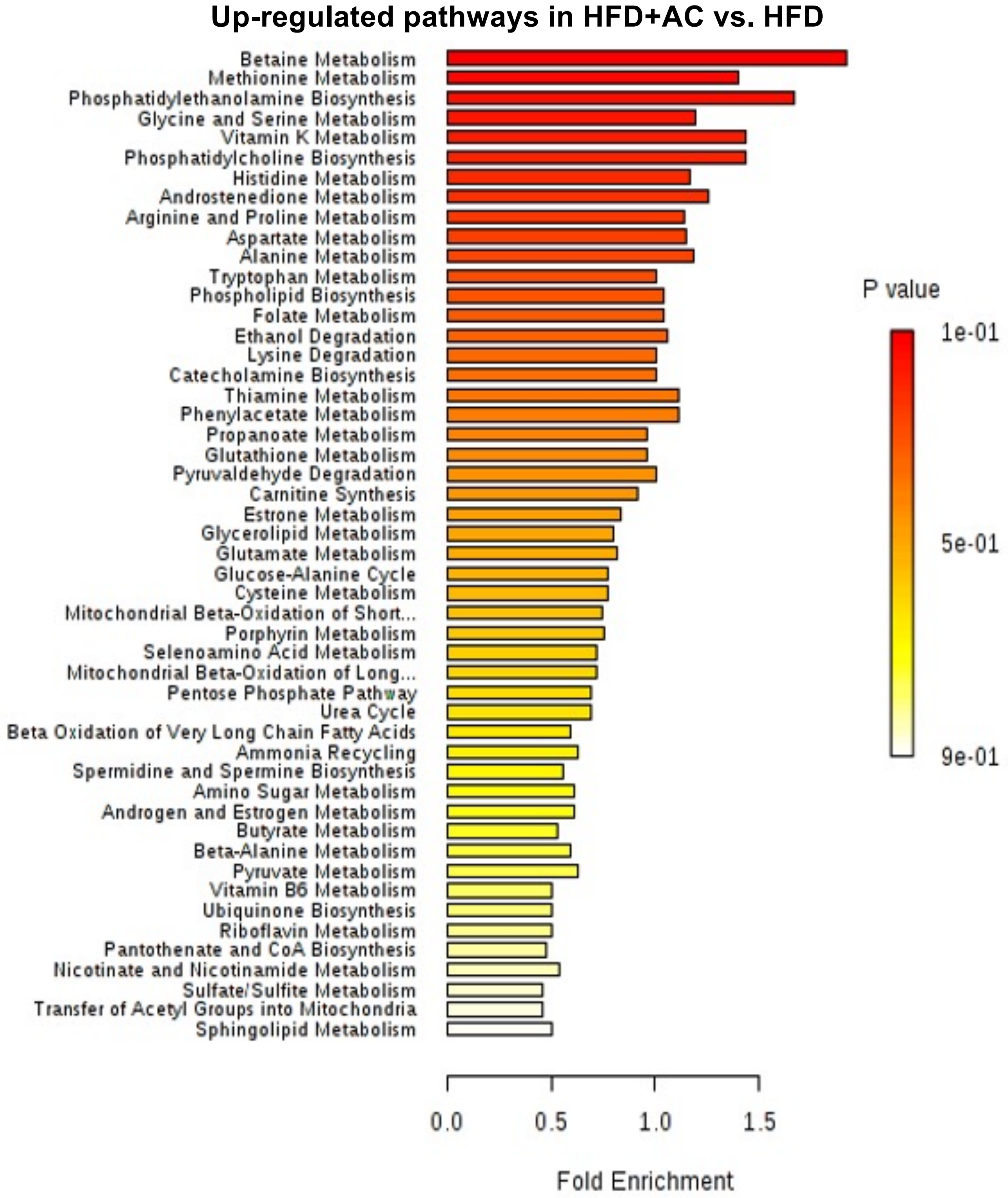
Up-regulated pathways in HFD+AC vs. HFD
Betaine Metabolism
Methionine Metabolism
Phosphatidylethanolamine Biosynthesis
Glycine and Serine Metabolism
Vitamin K Metabolism
Phosphatidylcholine Biosynthesis
Histidine Metabolism
Androstenedione Metabolism
Arginine and Proline Metabolism
Aspartate Metabolism
Alanine Metabolism
Tryptophan Metabolism
Phospholipid Biosynthesis
Folate Metabolism
Ethanol Degradation
Lysine Degradation
Catecholamine Biosynthesis
Thiamine Metabolism
Phenylacetate Metabolism
Propanoate Metabolism
Glutathione Metabolism
Pyruvaldehyde Degradation
Carnitine Synthesis
Estrone Metabolism
Glycerolipid Metabolism
Glutamate Metabolism
Glucose-Alanine Cycle
Cysteine Metabolism
Mitochondrial Beta-Oxidation of Short...
Porphyrin Metabolism
Selenoamino Acid Metabolism
Mitochondrial Beta-Oxidation of Long...
Pentose Phosphate Pathway
Urea Cycle
Beta Oxidation of Very Long Chain Fatty Acids
Ammonia Recycling
Spermidine and Spermine Biosynthesis
Amino Sugar Metabolism
Androgen and Estrogen Metabolism
Butyrate Metabolism
Beta-Alanine Metabolism
Pyruvate Metabolism
Vitamin B6 Metabolism
Ubiquinone Biosynthesis
Riboflavin Metabolism
Pantothenate and CoA Biosynthesis
Nicotinate and Nicotinamide Metabolism
Sulfate/Sulfite Metabolism
Transfer of Acetyl Groups into Mitochondria
Sphingolipid Metabolism
P value
1e-01
5e-01
9e-01
0.0
0.5
1.0
1.5
Fold Enrichment

Figure S5B, Continued

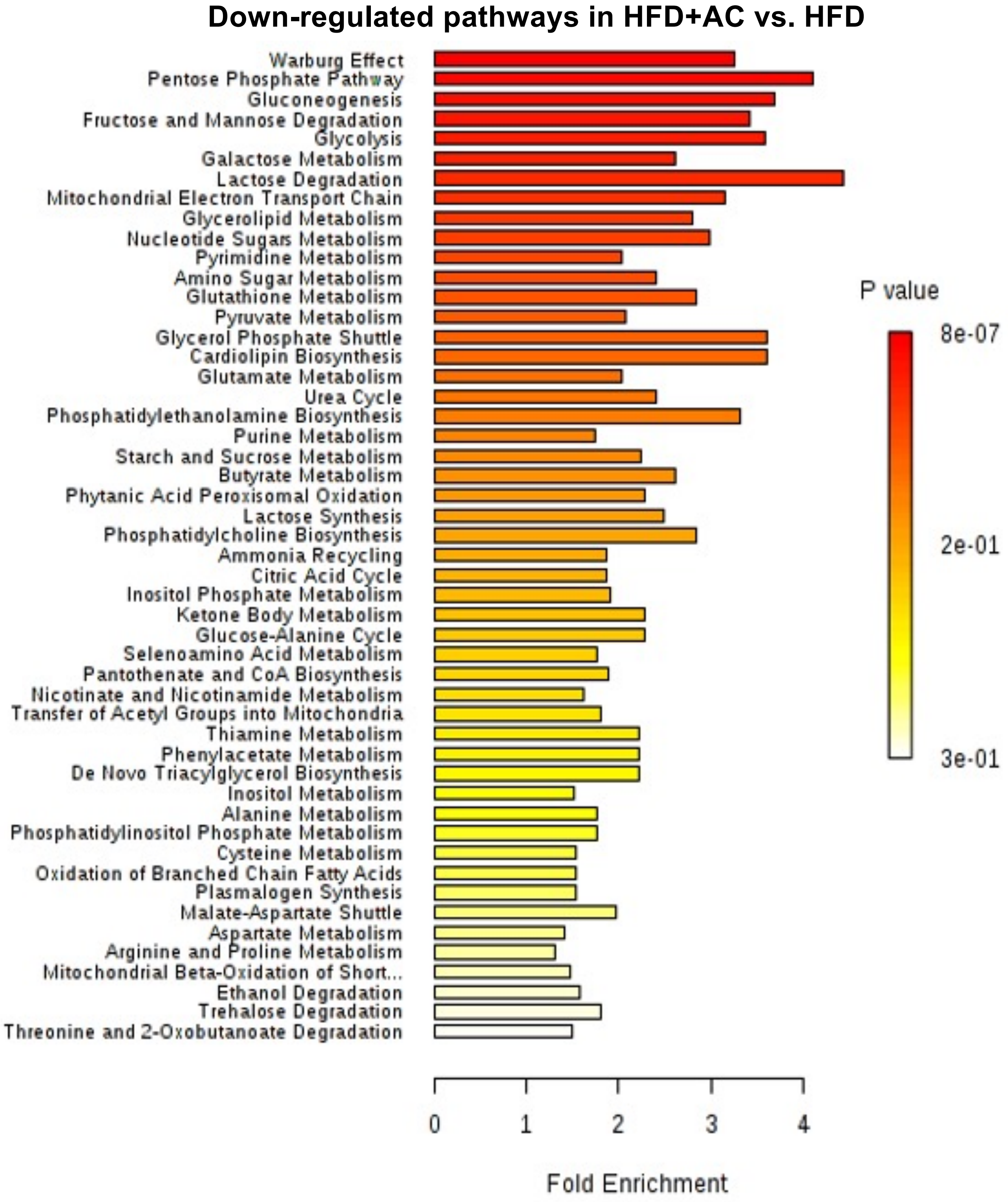

Figure S5B Alterations in pathways from hepatic metabolite levels measured by untargeted metabolomics. Pathways derived from the hepatic metabolites whose levels were significantly altered in the HFD+AC261066/HFD (n=4 per group with 2 repeats). HFD+AC261=HFD+AC261066.

Figure S5C

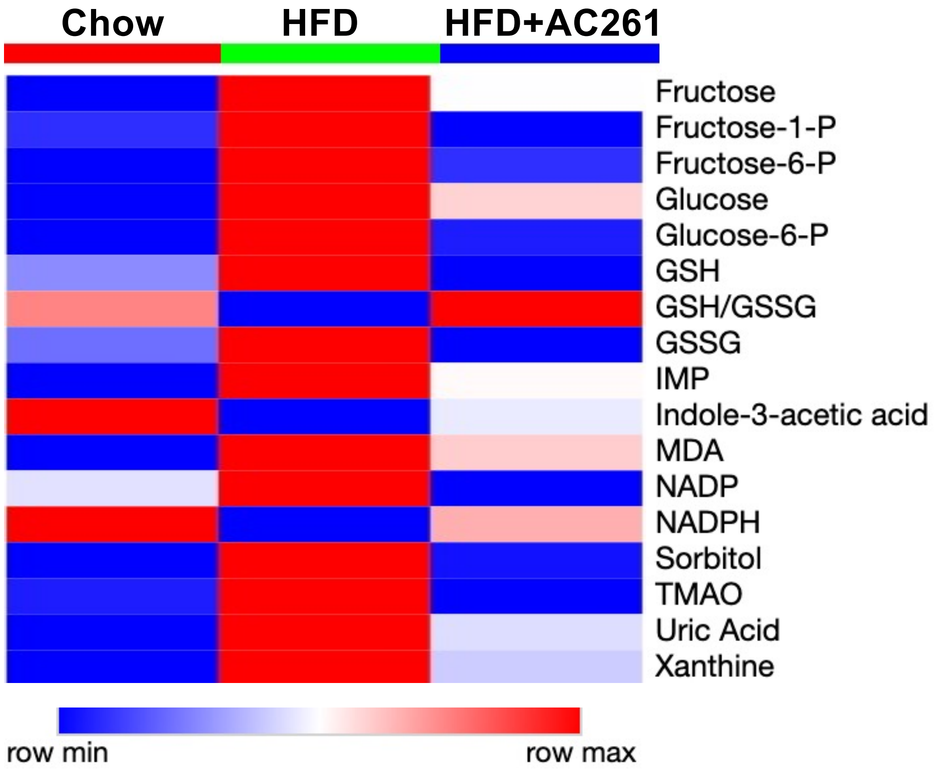
Chow
HFD
HFD+AC261
Fructose
Fructose-1-P
Fructose-6-P
Glucose
Glucose-6-P
GSH
GSH/GSSG
GSSG
IMP
Indole-3-acetic acid
MDA
NADP
NADPH
Sorbitol
TMAO
Uric Acid
Xanthine
row min
row max

Figure S5C Alterations in hepatic metabolite levels measured by untargeted metabolomics. Heatmap showing the levels of selected metabolites related to fructose metabolism and oxidative stress with altered statistically in the HFD/chow and the HFD+AC261066/HFD. The order of the metabolites is alphabetically. HFD+AC261=HFD+AC261066 (n=4 per group with 2 repeats).

Figure S6

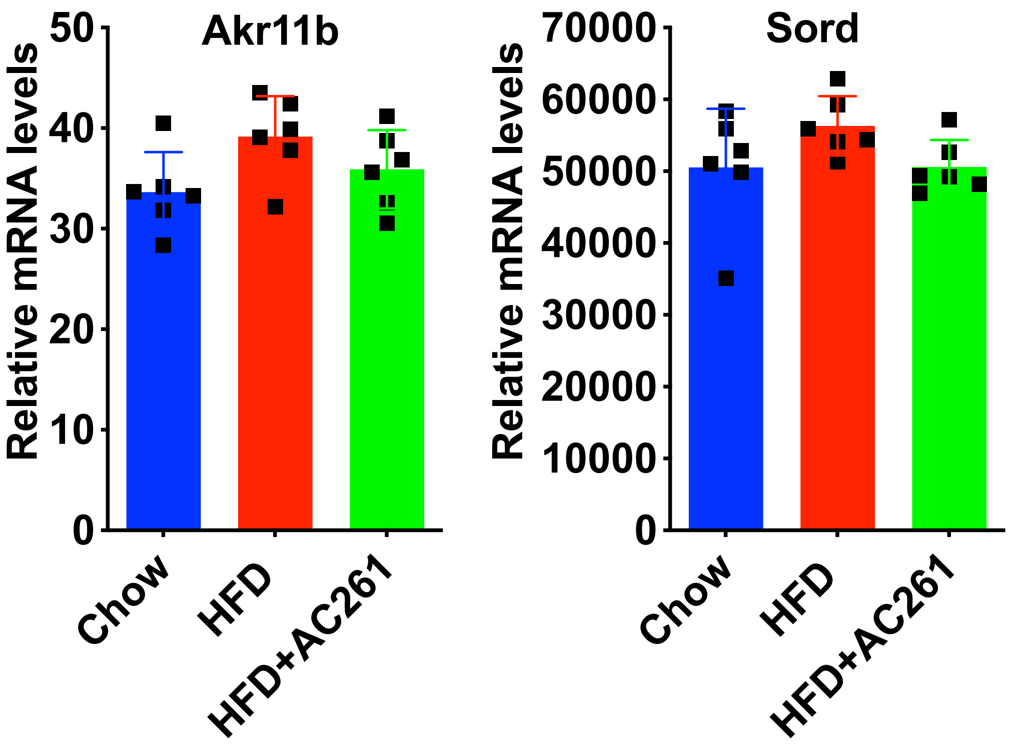
Akr11b
Relative mRNA levels
0
10
20
30
40
50
Chow
HFD
HFD+AC261
Sord
Relative mRNA levels
0
10000
20000
30000
40000
50000
60000
70000
Chow
HFD
HFD+AC261

Figure S6 Comparison of mRNA levels of two enzymes in the sorbitol (polyol) pathway from the RNA-seq data (n=6 per group)**.** The y axes (relative mRNA levels) are differentially expressed gene (DEG) transcript levels. HFD+AC261=HFD+AC261066.

Figure S7

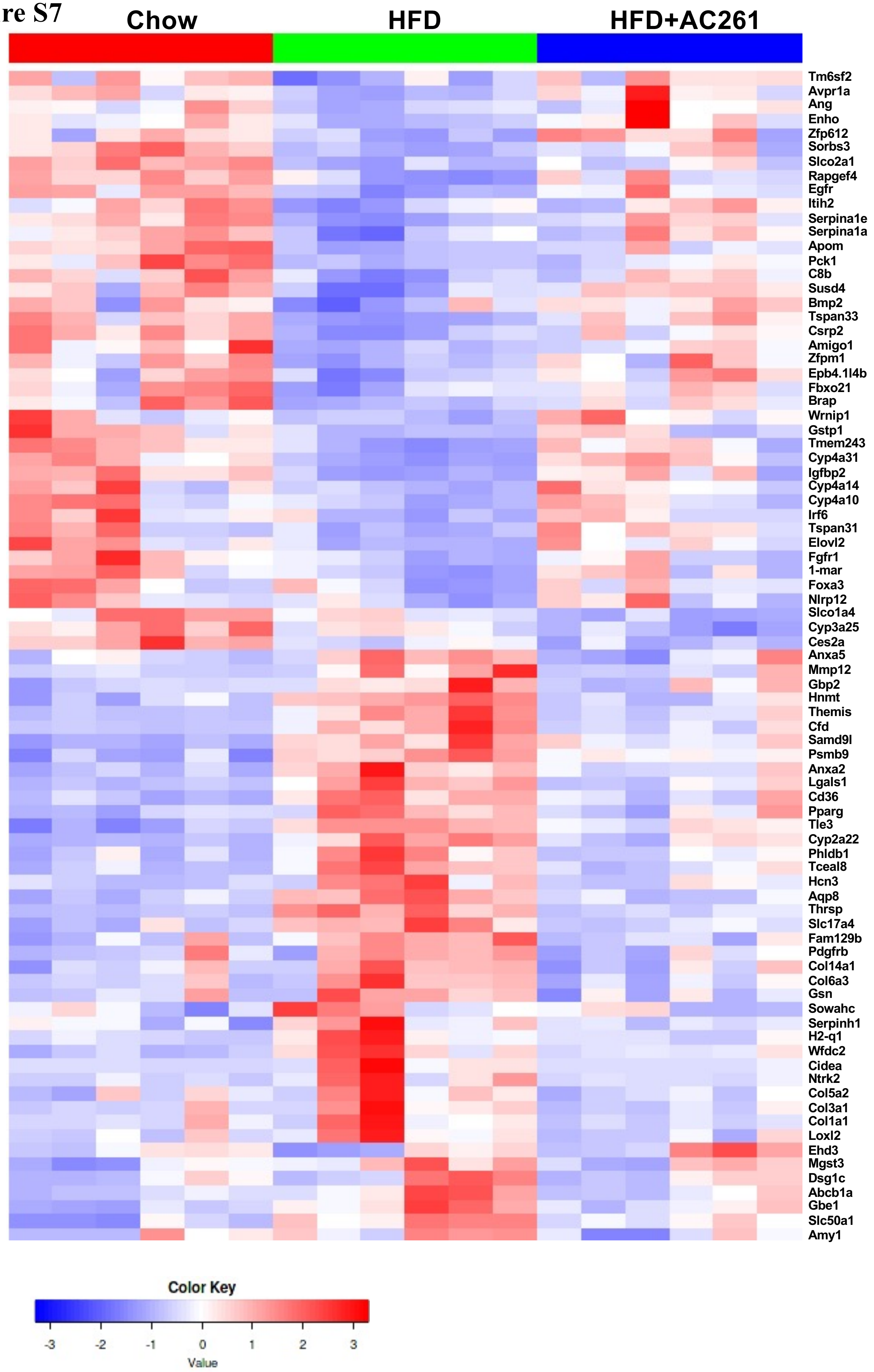
Chow
HFD
HFD+AC261
Tm6sf2
Avpr1a
Ang
Enho
Zfp612
Sorbs3
Slco2a1
Rapgef4
Egfr
Itih2
Serpina1e
Serpina1a
Apom
Pck1
C8b
Susd4
Bmp2
Tspan33
Csrp2
Amigo1
Zfpm1
Epb4.1l4b
Fbxo21
Brap
Wrnip1
Gstp1
Tmem243
Cyp4a31
Igfbp2
Cyp4a14
Cyp4a10
Irf6
Tspan31
Elovl2
Fgfr1
1-mar
Foxa3
Nlrp12
Slco1a4
Cyp3a25
Ces2a
Anxa5
Mmp12
Gbp2
Hnmt
Themis
Cfd
Samd9l
Psmb9
Anxa2
Lgals1
Cd36
Pparg
Tle3
Cyp2a22
Phldb1
Tceal8
Hcn3
Aqp8
Thrsp
Slc17a4
Fam129b
Pdgfrb
Col14a1
Col6a3
Gsn
Sowahc
Serpinh1
H2-q1
Wfdc2
Cidea
Ntrk2
Col5a2
Col3a1
Col1a1
Loxl2
Ehd3
Mgst3
Dsg1c
Abcb1a
Gbe1
Slc50a1
Amy1
Color Key
-3
-2
-1
0
1
2
3
Value

Figure S7 Heatmap showing the common mouse NASH related genes (Xiong et al., 2017) altered statistically (q<0.1) in the HFD/chow and the HFD+AC261066/HFD. The order of the transcripts is based on the data of HFD+AC261/HFD, *i.e.* transcripts increases in HFD+AC261/HFD first, followed by transcripts decreases in HFD+AC261/HFD. HFD+AC261=HFD+AC261066 (n=6 per group).

Figure S8

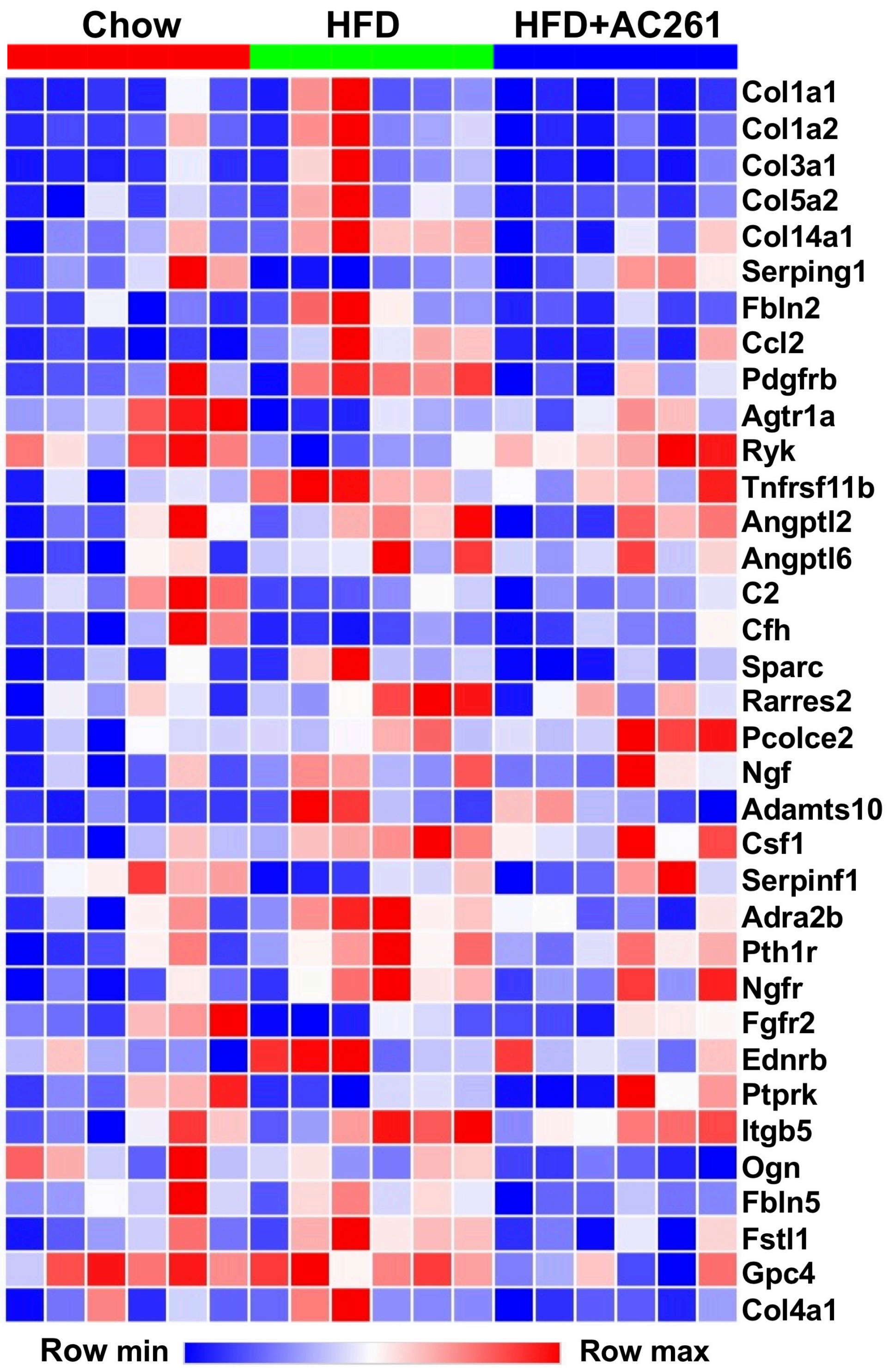

Chow
HFD
HFD+AC261
Col1a1
Col1a2
Col3a1
Col5a2
Col14a1
Serping1
Fbln2
Ccl2
Pdgfrb
Agtr1a
Ryk
Tnfrsf11b
Angptl2
Angptl6
C2
Cfh
Sparc
Rarres2
Pcolce2
Ngf
Adamts10
Csf1
Serpinf1
Adra2b
Pth1r
Ngfr
Fgfr2
Ednrb
Ptprk
Itgb5
Ogn
Fbln5
Fstl1
Gpc4
Col4a1
Row min
Row max

Figure S8 Heatmap analyses showing common transcripts significantly altered in the HFD/chow and the HFD+AC261066/HFD that are involved in HSC secretome (Xiong et al., 2017). The order of the transcripts is based on the data of HFD+AC261/HFD*, i.e.* transcripts decreases in HFD+AC261/HFD. HFD+AC261=HFD+AC261066 (n=6 per group).

Figure S9A

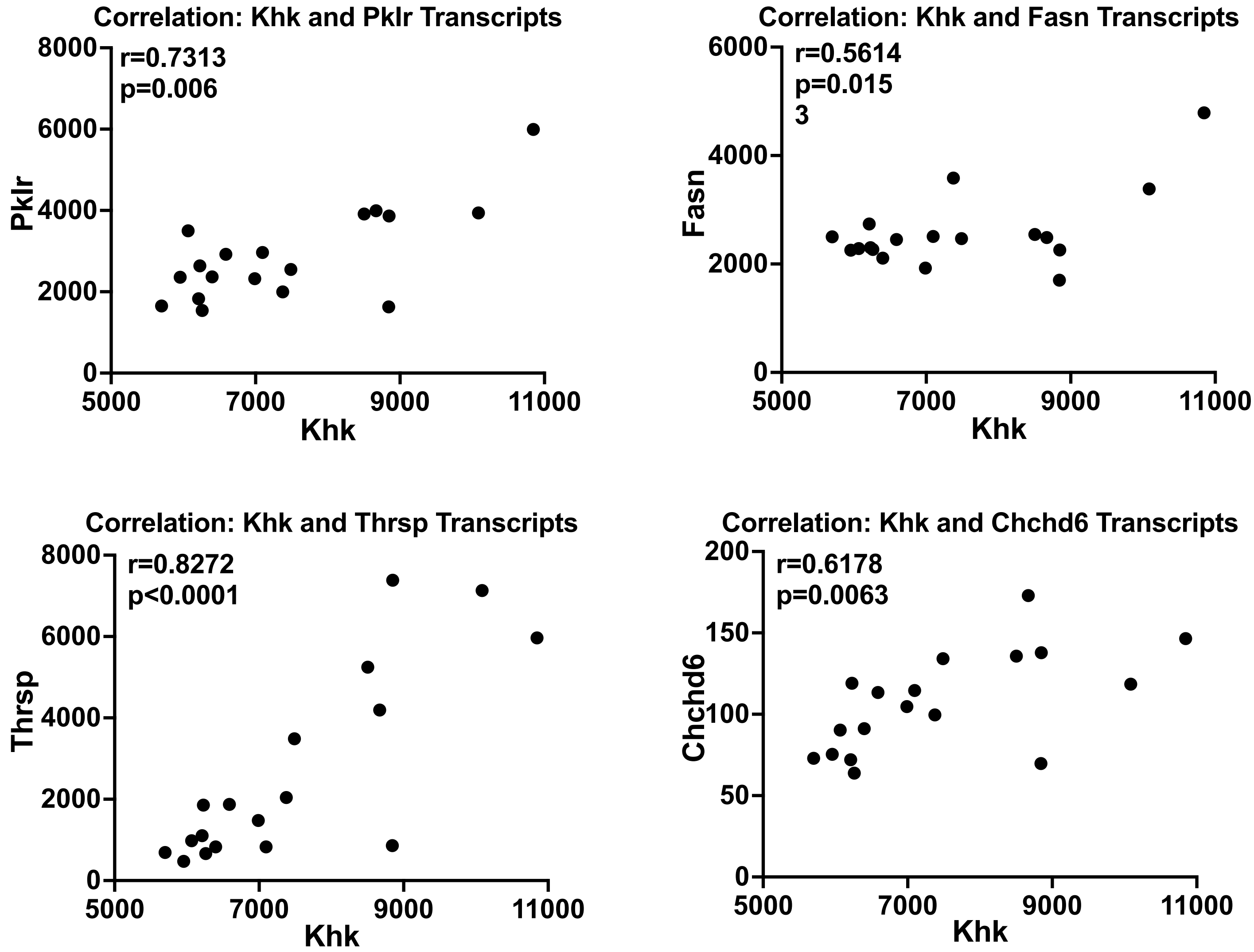
Correlation: Khk and Pklr Transcripts
r=0.7313
p=0.006
Pklr
Khk
Correlation: Khk and Fasn Transcripts
r=0.5614
p=0.015
3
Fasn
Khk
Correlation: Khk and Thrsp Transcripts
r=0.8272
p<0.0001
Thrsp
Khk
Correlation: Khk and Chchd6 Transcripts
r=0.6178
p=0.0063
Chchd6
Khk

Figure S9B

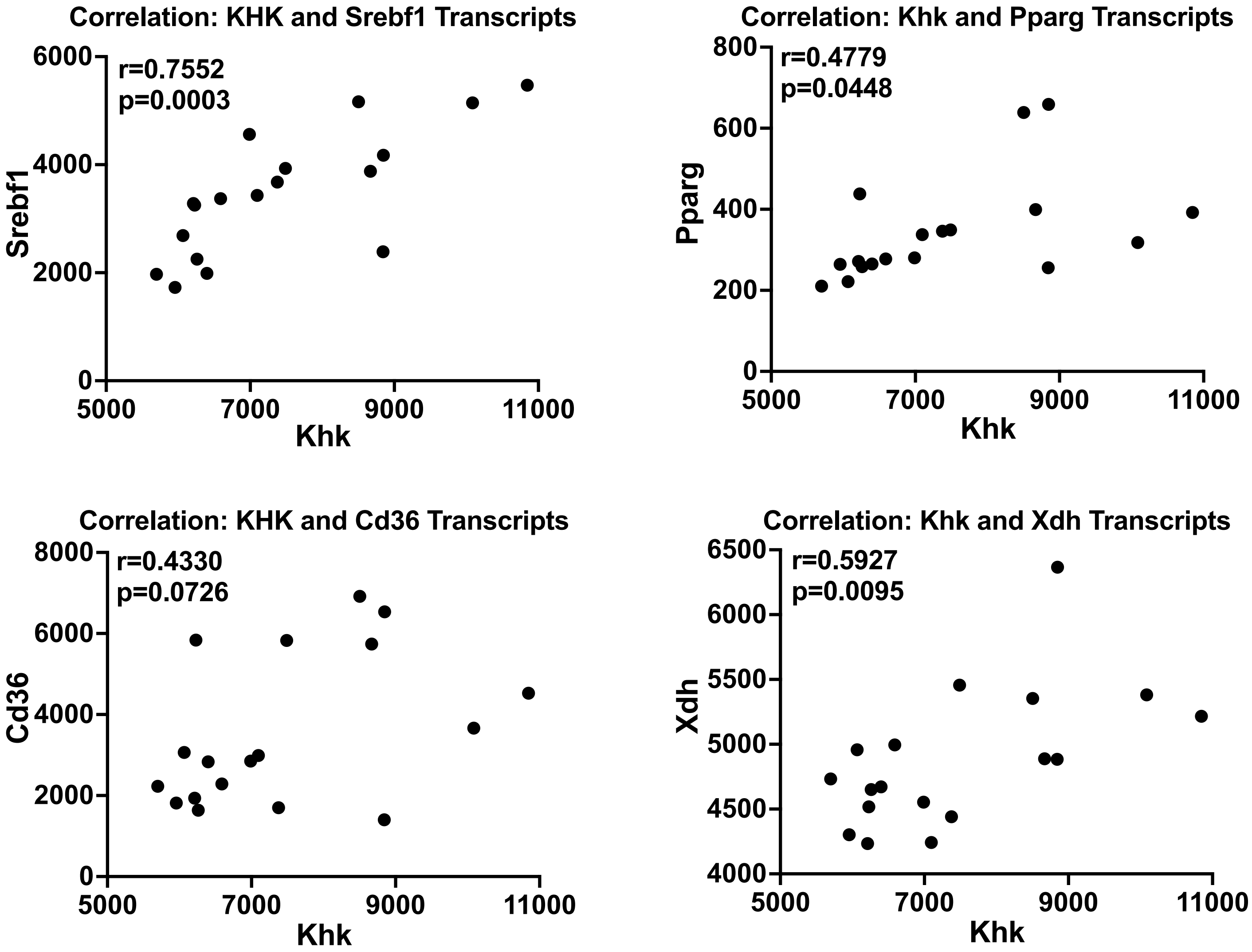
Correlation: KHK and Srebf1 Transcripts
r=0.7552
p=0.0003
Srebf1
Khk
Correlation: Khk and Pparg Transcripts
r=0.4779
p=0.0448
Pparg
Khk
Correlation: KHK and Cd36 Transcripts
r=0.4330
p=0.0726
Cd36
Khk
Correlation: Khk and Xdh Transcripts
r=0.5927
p=0.0095
Xdh
Khk

Figure S9C

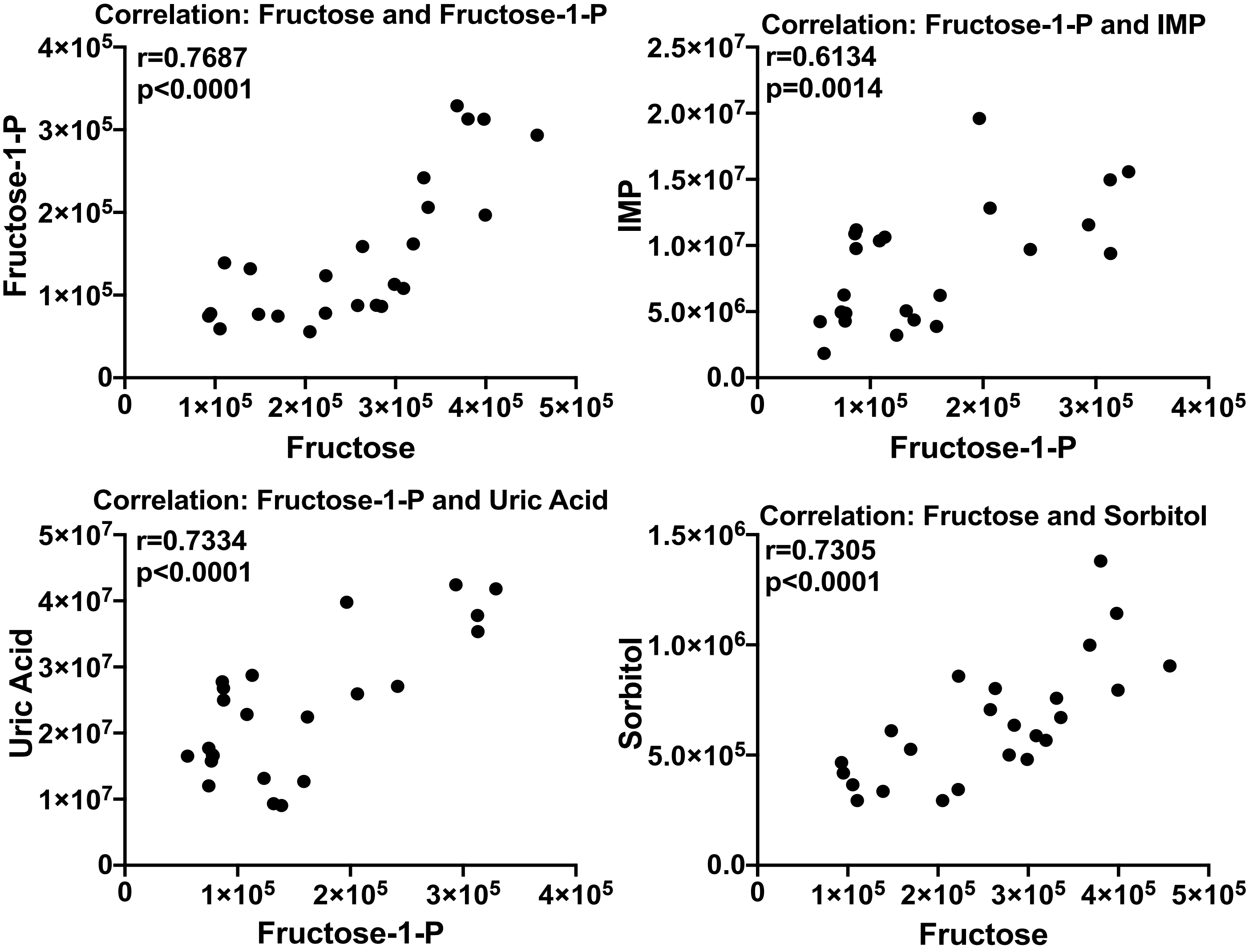
Correlation: Fructose and Fructose-1-P
r=0.7687
p<0.0001
Fructose-1-P
Fructose
Correlation: Fructose-1-P and IMP
r=0.6134
p=0.0014
IMP
Fructose-1-P
Correlation: Fructose-1-P and Uric Acid
r=0.7334
p<0.0001
Uric Acid
Fructose-1-P
Correlation: Fructose and Sorbitol
r=0.7305
p<0.0001
Sorbitol
Fructose

Figure S9 Pearson correlation analyses between transcript and metabolite levels. Transcript and metabolite levels in all groups were used for the analyses. R, correlation coefficient. Statistical significance is defined as $p<0.05$.
